# Beyond reward learning deficits: Exploration-exploitation instability reveals computational heterogeneity in value-based decision making in early psychosis

**DOI:** 10.1101/2025.04.29.25326698

**Published:** 2025-05-01

**Authors:** Cathy S. Chen, Evan Knep, Veldon-James Laurie, Olivia Calvin, R. Becket Ebitz, Melissa Fisher, Michael-Paul Schallmo, Scott R. Sponheim, Matthew V. Chafee, Sarah R. Heilbronner, Nicola M. Grissom, A. David Redish, Angus W. MacDonald, Sophia Vinogradov, Caroline Demro

**Affiliations:** 1 Department of Psychiatry and Behavioral Sciences, University of Minnesota, Minneapolis, Minnesota, United States; 2 Department of Psychology, University of Minnesota, Minneapolis, Minnesota, United States; 3 Department of Neurosciences, University of Montréal, Québec, Canada; 4 Department of Neuroscience, University of Minnesota, Minneapolis, Minnesota, United States; 5 Department of Neurosurgery, Baylor College of Medicine, Houston, Texas, United States; 6 Minneapolis VA Health Care System, Minneapolis, MN, United States

**Keywords:** psychosis, exploration-exploitation tradeoff, value-based decision making, reinforcement learning, uncertainty intolerance, computational psychiatry

## Abstract

Psychosis spectrum illnesses are characterized by impaired goal-directed behavior and significant neurophysiological heterogeneity. To investigate the neurocomputational underpinnings of this heterogeneity, 75 participants with Early Psychosis (EP) and 68 controls completed a dynamic decision-making task. Consistent with prior studies, EP exhibited more choice switching, not explained by reward learning deficits, but instead by increased transition to exploration from exploitation. Bayesian modeling implicated elevated uncertainty intolerance and decision noise as independent contributors to suboptimal transition dynamics across individuals, which identified three computational subtypes with unique cognitive and symptom profiles. Replicating prior studies, a high decision-noise subtype emerged showing learning deficits and worse negative symptoms; our analyses further uncovered a normative subtype with worse mood symptoms and a novel uncertainty-intolerance subtype with higher hospitalization rates. These specific microcognitive disruptions underlying the distinct neurocomputational subtypes are individually measurable and may have the potential for targeted interventions.

## Introduction

Psychosis spectrum illnesses exhibit significant clinical and cognitive heterogeneity in symptoms, illness trajectory, and treatment response^1^. While impairments in goal-directed behavior are common, their specific nature and severity can vary widely across individuals. This heterogeneity poses challenges for understanding the underlying pathophysiology and developing more efficacious individualized treatment strategies. Computational psychiatry has emerged as a promising framework for parsing interindividual variability beyond observed behavior^2^. Modeling of trial-by-trial behavior can reveal distinct latent strategies used in goal-directed decision making and offer insights into individualized cognitive profiles^3^. Computational modeling of “microcognitive” decision-making mechanisms may illuminate why goal-directed behavior is affected in different ways across people with psychosis, potentially leading to new avenues for targeted interventions.

Adaptive goal-directed behavior requires balancing the decision to sustain known rewarding actions against the decision to try new options, often referred to as “the exploration-exploitation tradeoff.” Disruptions in exploration-exploitation behavior have been frequently reported in psychosis spectrum illness, most notably excessive choice switching and less persistent exploitation^4–14^. Some studies have implicated reward learning deficits such that patients failed to learn which choices were rewarding, leading to excessive switching^11–13^. Other studies point to elevated decision noise or random exploration, meaning that patients’ choices were more variable and less driven by expected value^15^. But the exact contributions made by each of these potential mechanisms has remained unexplained.

More recent computational modeling approaches have identified latent microcognitive parameters - such as learning rate, random exploration, and directed exploration - that contribute to alterations in exploration-exploitation tradeoff in psychosis patients^15,16^. Based on these computational parameters, prior studies have revealed meaningful computational phenotypes characterized by different underlying impairments. For example, Waltz et al. (2020) identified a subtype of schizophrenia patients exhibiting extreme ambiguity aversion (i.e. extreme avoidance of uncertain options), which correlated with poorer working memory and lower IQ. Similarly, Whitton et al. (2024) uncovered computational phenotypes with varied effort allocation strategies in decision making that were linked to symptom severity and functioning among individuals with schizophrenia spectrum illnesses^17^. One subtype, which failed to use reward value and probability information to guide effort allocations, was associated with more severe positive and negative symptoms along with poorer cognitive and community functioning. Although Whitton et al. did not directly investigate the exploration–exploitation tradeoff, their findings highlighted key microcognitive processes that integrate reward and uncertainty for adaptive decision making. These findings demonstrate the potential for computational modeling to parse decision-making profiles within the psychosis spectrum, which would offer critical insights into underlying pathophysiology and potential intervention targets.

Despite these computational advancements, most prior value-based decision making research has focused on reward learning under relatively stable reward contingencies, with limited attention to how decisions are made under dynamic and uncertain environments that require actively navigating the exploration-exploitation tradeoff. As a result, it remains unclear how individuals adapt their strategies when reward contingencies change in volatile or unpredictable ways. By explicitly modeling decisions within a task where reward contingencies change randomly and unpredictably, our study addresses this knowledge gap by providing a more refined analysis of how individuals transition between exploration and exploitation based on observed feedback as well as perceived changes in the environment. Furthermore, our three-choice task design allows us to parse out value-driven, information-driven and random decisions, providing more fine-grained insights into microcognitive processes underlying individual variability in decision-making strategies. We predicted, based on prior research, that EP as a group would exhibit excessive switching between choices compared to controls. By leveraging the unique features of our task, we sought to parse out the distinct microcognitive processes underlying the explore-exploit transitions, and we hypothesized that we would identify EP subtypes who share this general over-exploration pattern yet differ in the specific cognitive mechanisms driving transitions towards over-exploration.

We recruited 75 participants with early psychosis (EP) and 68 neurotypical comparison participants to perform an open-ended three-armed restless bandit task designed to evoke interindividual variability in adaptive decision-making strategies in changing environments^18–20^. We used a latent state model and a Bayesian learning model to examine individual differences in utilization of explore/exploit strategies and the microcognitive factors guiding transitions between these strategies. We found that EP showed similar overall reward acquisition as the comparison group, but diverging underlying strategies. Overall, and consistent with previous work, EP switched choices more frequently; however, computational modeling revealed that this resulted from suboptimal transitions into exploration. These suboptimal transitions to exploration correlated with poorer real-world functioning. Computational modeling also uncovered uncertainty intolerance and decision noise as two distinct and independent computational processes that contribute to the transitions between exploration and exploitation. Higher decision noise correlated with more severe negative symptoms, while no significant associations between uncertainty intolerance and clinical features were observed among EP on the group level. Hierarchical clustering revealed meaningful computational subtypes within the EP group, characterized by distinct contributors to explore/exploit transition dynamics and cognitive features. Each subtype was associated with unique symptom profiles, further dissecting the heterogeneity. Together, our findings extend prior work by identifying how distinct microcognitive processes can each contribute independently to changes in decision making behavior and potentially shed light on distinct neurophysiological mechanisms and individualized treatment needs.

## Results

Participants were recruited as part of a larger longitudinal neuroimaging study of early psychosis at the University of Minnesota. A total of 143 participants (75 participants with Early Psychosis (EP) and 68 control participants with no history of psychosis, Table 1) completed a 200-trial three-armed restless Bandit task, during the first session of the study, and 124 of those participants (*n*=61 participants with EP; *n*=63 control participants) completed the same task (with 300 trials) during a second session approximately 3 weeks later. See Table 1 for participant characteristics and inclusion and exclusion criteria in the Methods section.

**Figure 1.**
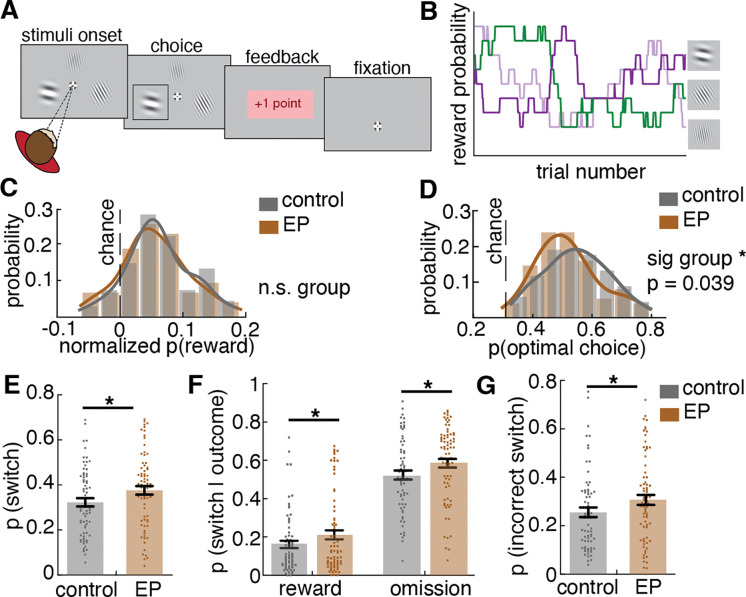
Participants with Early Psychosis were over switching, regardless of the outcome and the most optimal choice in the restless Bandit Task. **A**) task schematic. A three-armed restless Bandit task, in which participants chose from one of three gabor patch stimuli and received feedback of reward (+1 point or +0 points). Reward was probabilistic. Participants completed the restless Bandit task at two sessions, approximately three weeks apart. Data from both timepoints were used in analyses. **B**) An example of dynamic reward contingency where the probability of reward associated with each gabor stimulus changed slowly, independently, and randomly over time, **C**) Average reward acquisition normalized by chance level. **D**) Average probability of choosing the optimal choice (highest payoff choice). EPs were less likely to choose the optimal choice. **E**) Average probability of switching to a different choice, **F**) Average probability of switch given a rewarded or reward omission outcome in the previous trial (i.e. win switch and lose switch). **G**) Average probability of incorrect switch (switching away from an optimal choice). Since there were no prominent session effects, only session 1 data is shown here for clarity. Session 2 results can be found in Extended Data Figure 1. * indicates p < 0.05. Graphs depict mean ± SEM across participants.

The three-armed restless Bandit task is a dynamic variant of a classic stochastic reinforcement learning task (“Bandit task”). During each trial, participants chose among three Gabor patches, each of which was associated with some probability of giving +1 point (reward) or +0 points (no reward) (Figure 1A). The reward probability of each choice changed randomly and independently over time (Figure 1B). Participants had to sample across choices and learn from the outcome. The goal of the task was to maximize the points earned for which they received a monetary bonus. The dynamic reward contingency of the task naturally encouraged participants to balance between exploiting what has been learned to be rewarding currently and exploring alternatives that could become more valuable at any time. Participants were told that the reward probability associated with each image would change slowly and randomly over the course of the task, but they were not given further instruction on how or when they should start exploring, allowing for maximum individual differences in divergent decision-making strategies employed to solve the task. Prior to the task, participants completed 25 practice trials during which the reward probability of three choices was fixed at [0.7, 0.3, 0.2]. Two criteria needed to be met before proceeding with the task: 1. Participants need to score above chance (at least 11 of 25 points), 2. Participants need to switch choices at least twice. These criteria ensure that participant behavior is sufficiently flexible to explore options while also able to sustain reward. The practice training helps the participant to understand that the task is stochastic, such that even the best choice does not always provide a reward, and that they need to do both exploring and exploiting to succeed in this task.

We used linear mixed effects models to test for group differences across the two data acquisition sessions. Since results from both sessions were qualitatively similar, figures in the main text only show results for the first session for clarity. The results for the second session are shown in the Extended data figures. All statistical models included covariates for gender and chance of reward on the restless Bandit task. Gender was included as a covariate in the analyses because the proportions of gender identity differed significantly across groups. Since the reward contingency (“walk”) was generated and assigned to participants randomly, the chance level of reward differed slightly from session to session (mean chance level of reward across all three arms was constrained to be in the range of 48%–52%). Therefore, the chance level of getting a reward was also included as a covariate in all statistical models.

### Task performance was similar across participants with early psychosis and controls

**Extended Data Figure 1.**
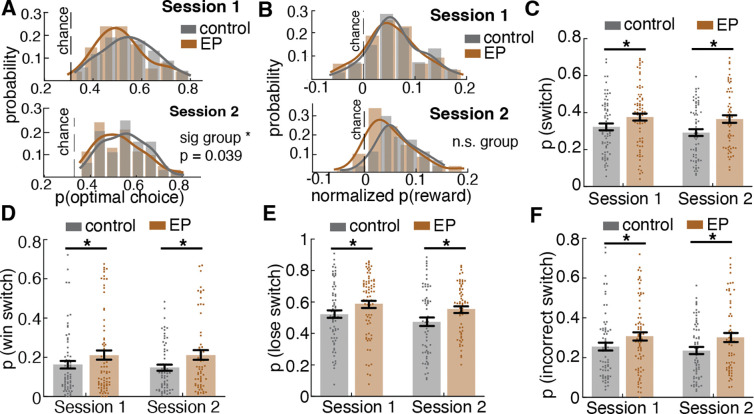
Task performance and switching behaviors in the restless bandit task across Session 1 and Session 2. Related to Figure 1. **A**) Average probability of choosing the optimal choice (highest payoff choice), **B**) Average reward acquisition normalized by chance level, **C**) Average probability of switching to a different choice, **D**) Average probability of switch after a rewarded trial (win switch), **E**) Average probability of switch after a reward omission trial (lose switch), **F**) Average probability of incorrect switch (switching away from an optimal choice).

In order to determine whether all participants sufficiently understood and engaged with the task, as opposed to responding randomly, we first examined overall task performance. Due to the unique “restless” feature of the task, performance is best measured by the amount of reward obtained over the session relative to random choosing, and by the number of “optimal” decisions. We examined reward acquisition by calculating the average probability of obtaining a reward over the whole session relative to choosing randomly. We found no significant group difference in reward acquisition between participants with EP and control participants (*F*(1,141)=2.18, *p*=.142). Because the reward contingency changed randomly, the probability of reward available over the session could differ across participants. We therefore compared the probability of reward obtained relative to the average probability of reward if chosen randomly (chance level). Participants with EP and control participants both performed significantly better than chance in session 1 (Figure 1C; one sample t-test, control: *t*(67) = 9.86, *p*<.0001, EP: *t*(74) = 9.0, *p*<.0001) and session 2 (Extended Data Figure 1: control: *t*(62) = 12.41, *p*<.0001, EP: *t*(60) = 8.23, *p*<.0001), with no significant group difference across either session (*F*(1,141)=2.18, *p*=.142). Additional evidence of equal task engagement was that we found no group differences in the number of trials completed in either session (*F*(1,141)=1.68, *p*=.197), nor in stimulus position bias (repeated key press) (*p*>.302 at session 1, *p*>.263 at session 2) and only a non-significant trend toward a group difference in response time (time elapsed between stimulus onset and key press for response), consistent with moderate slowing on timed tests among people with psychosis ^21^ (*F*(1,141)=3.42, *p*=.067). To further examine overall task performance, we calculated the probability of selecting the optimal choice, which is defined as choosing the highest payoff choice across all choices for each given trial. It is important to note that which choice was optimal was never directly revealed to the subject — it had to be inferred from sequences of rewarded and non-rewarded experiences. We found a significant group difference such that participants with EP had lower likelihood of selecting the optimal choice on average compared to control participants (Figure 1D; *F*(1,141)=4.32, *p*=.039).

Together, these results indicate that both groups showed good task engagement and understanding of reward contingencies to achieve better-than-chance rewards. While participants with EP were less accurate at choosing the most optimal choice at any given trial, their reward acquisition performance across all trials was comparable to control participants. The similar response time across groups suggests that the less “optimal” choice selection in participants with EP was not a speed accuracy tradeoff^22^.

### Participants with early psychosis switched choices more often than controls regardless of the outcome and the optimal choice

In a restless bandit environment, similar levels of reward acquisition performance can be achieved using different underlying strategies, due to the stochastic and dynamic nature of the reward contingency. For example, a high-switching strategy might catch onto a high value choice by chance sooner, while a low-switching strategy is more robust to transient non-rewarded trials arising from the probabilistic nature of the task. Therefore, it is important to examine how individuals were making decisions trial-by-trial. First, we examined overall stay (repeating a previous choice) and switch (changing to a different choice than previous) probabilities to understand how individuals assessed stability versus change in their environments. Despite comparable reward acquisition, participants with EP showed increased switching behavior relative to control participants (Figure 1E; *F*(1,141)=7.67, *p*=.006). Specifically, compared to controls, participants with EP were more likely to switch away from a choice after receiving a reward (win-switch; Figure 1F, *F*(1,141)=3.94, *p*=.049), to switch away from a choice if it was not rewarded (reward omission-switch; Figure 1F, *F*(1,141)=7.53, *p*=.007), and also more likely to switch away from the optimal option defined as the highest payoff option (incorrect switch; Figure 1G, *F*(1,141)=5.17, *p*=.024). Together, these results suggest that participants with EP engaged in more switching behavior regardless of the outcome, resulting in being more likely to switch away from an optimal choice and making fewer optimal choices over time. This is consistent with our hypothesis and the prior literature^4–14^.

### Participants with early psychosis explored more than controls as a result of instability in exploration-exploitation strategy

Although our finding of increased switching in EP is consistent with prior literature^11,14,23^, a question that remains unanswered is why participants with early psychosis switched more often than control participants. One possibility is that participants with EP had a constant higher probability of switching after repeating for a certain amount of time (i.e., a faster time constant for switching). Another possibility is that all participants used multiple strategies, with EP over-switching because they favored a high-switching strategy.

**Figure 2.**
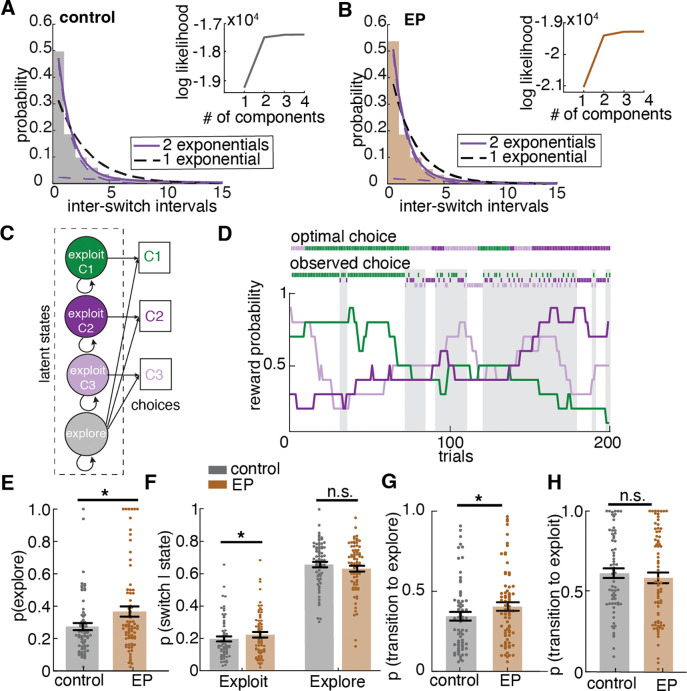
Participants with Early Psychosis over-explored due to a higher probability of transitioning into the explore strategy state, compared to controls. **A, B**) The distribution of inter-switch intervals and mixture model fit for control participants (A) and participants with EP (B). A single probability of switching would produce exponentially distributed inter-switch intervals. Black dotted line - the maximum likelihood fit for a single discrete exponential distribution. Purple solid line - a mixture of two exponential distributions, with each component distribution in dotted purple line. The two components reflect one fast-switching regime and one persistent regime. The inset is the log likelihood of mixtures of 1–4 exponential distributions. A clear elbow effect can be observed at 2 components. **C**) Hidden Markov model (HMM) model structure. The HMM models exploration and exploitation as latent strategy states underlying observed choices. This model includes an exploitation state for each choice, and an exploration state. **D**) Reward probabilities (colored lines), observed choices (lower dots), optimal choices defined by the highest payoff (upper dots), and HMM-labeled exploratory choices (shaded area) in an example session of 200 trials. **E**) Participants with Early Psychosis (EP) had increased exploration. **F**) Participants with EP had higher probability of switching during exploit strategy state. **G**) No group difference in the probability of switching during explore strategy state. **H**) EP had a higher probability of transitioning to explore strategy state (fitted transition matrix parameter), resulting in over exploration in EP. **I**) No group difference in the probability of transitioning to exploit strategy state. Since there were no session effects, only session 1 data is shown here for clarity. Session 2 results can be found in Extended Data Figure 2. * indicates p < 0.05. Graphs depict mean ± SEM across participants.

To examine whether participants’ choice behavior was characterized by a single strategy or multiple strategies with different time constants for switching, we analyzed the temporal structure of the choice sequences using a mixture model of exponential distributions. If choice behavior had a single underlying time constant for switching, or, in other words, some fixed probability of moving away from the current option at any given trial, then we would expect to see the inter-switch intervals (the number of consecutive repeated choices between switches) be exponentially distributed^24^. However, if there exist multiple time constants for switching in the choice sequence, the inter-switch intervals would be distributed as a mixture of multiple weighted exponential distributions. A mixture of two components - a fast-switching regime (putatively exploration) and a slow-switching regime (putatively exploitation) — provided the most parsimonious explanation for the choice behaviors in both control and EP groups (Figure 2A, 2B), consistent with the performance of non-human animals (mice^25,26^, and monkeys^19,25^) on equivalent tasks.

Given that the mixture model revealed two temporally distinct behavioral regimes on a group level, we turned to latent state modeling approaches to further examine the individual-level heterogeneity by modeling the fast-switching regime (putatively exploration) and slow-switching regime (putatively exploitation) as two latent strategy states, fitting them to each participant’s choice sequences. In previous research, the Hidden Markov model (HMM) approach to infer latent explore/exploit strategy has shown reliable behavioral and neural correlates of states across species^19,20,26–28^. To infer latent strategy (state) from observed choice sequences, we fit an HMM with exploration and exploitation as two separate latent strategy states emitting different patterns of choices, labeling each choice as explore or exploit. Exploit states yield repeated choices, whereas explore states can yield all three choices. Figure 2C illustrates the HMM latent states as well as the transition structure. Since the mixture model suggested two patterns of choice, our model consisted of two types of hidden states, providing different emission probabilities of choices, and the transition probability between states. Shown in Figure 2D is an example HMM labeling of choice, where the shaded area is the HMM-inferred exploration. During exploration bouts, choices were more distributed across all three options, whereas during exploitation bouts, repeated selection of a single choice was observed. The model-labeled strategy states were also associated with different win-stay lose-shift patterns, which capture the observed changing sensitivity to outcomes. We found a higher probability of win-stay during the exploit state than during the explore state (main effect of state, *F*(385)=137.73, *p*<.001) and higher probability of lose-shift during the explore state than during the exploit state (main effect of state, *F*(385)=2100.48, *p*<.001) in both groups, implicating adaptive outcome sensitivity in explore and exploit strategy state and supporting the face validity of HMM-state labels to capture meaningfully distinct latent strategies.

**Extended Data Figure 2.**
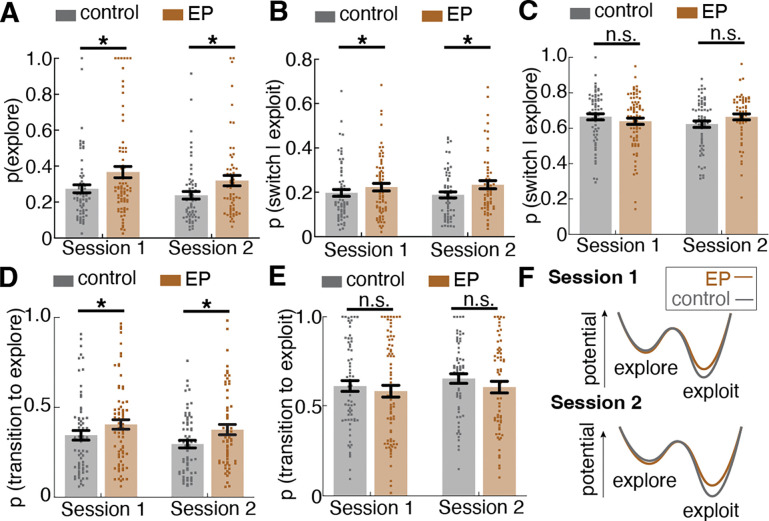
Hidden Markov Model (HMM)-inferred exploration and explore/exploit transition dynamics across Session 1 and Session 2. Related to Figure 2. **A**) Probability of exploration inferred from HMM, **B**) Probability of switching during exploit strategy state, **C**) Probability of switching during explore strategy state, **D**) Probability of transitioning to explore strategy state from exploit strategy state, **E**) Probability of transitioning to exploit strategy state from explore strategy state, **F**) Dynamic landscape of fitted HMMs for controls and EPs for session 1 (top) and session 2 (bottom). The model fit to EP had shallower and less stable exploit state, making it easier to transition out of exploiting a high value choice and start exploration too soon.

To examine how much a given person explored, we calculated the frequency of the exploratory choices labeled by the HMM fitted to each participant. The result suggests that participants with Early Psychosis (EP) explored more often than controls (Figure 2E, *F*(1,141)=7.08, *p*=.009). Specifically, EP had 36.7% ± 27.1% of trials labeled as exploration at session 1 and 32.0% ± 22.3% at session 2 whereas controls had 27.4% ± 18.3% of trials labeled as exploration at session 1 and 23.8% ± 16.6% at session 2 (Extended Data Figure 2). To test whether over-exploration reflects over-switching regardless of strategy state or over-switching during one specific strategy state, we examined the probability of switching in each strategy state. Participants with EP switched more during the exploitation strategy state (Figure 2F, F(1,138) = 5.53, p = .020). Surprisingly, there was no group difference in the switch rate during exploration (Figure 2G, F(1,141) = 0.03, p=.871), indicating a similar switching pattern during exploration for both controls and EP participants.

The higher switch rate out of the exploitation strategy state into the exploration strategy state in EP should be indicated by a difference in the transition dynamic between strategy states. Therefore, we examined the transition matrix describing the one-step transition probability within and between the explore and exploit states fit to individuals’ choice sequences. Both an increase in probability of transitioning to explore strategy state and a decrease in probability of transitioning to exploit strategy state can lead to an increase in overall exploration. The result revealed that the increased exploration in participants with EP was a result of a higher probability of transitioning from exploit to explore strategy states (Figure 2F, *F*(1,141)=8.32, *p*=.005). Further analysis confirmed the instability in the reward-driven exploit strategy state among EP (Extended Data Figure 2F, Supplemental Materials). Interestingly, when looking at the effect of outcome on strategy transition, we found that participants with EP were more likely to transition to an explore strategy after a rewarded trial than control participants (*F*(1,141)=8.72, *p*=.004), suggesting that loss avoidance does not fully explain this behavior. Of note, there were no group differences in the probability of transitioning from explore to exploit strategy, suggesting an intact ability to learn the value of choices during exploration and start committing to a favorable choice once it is found (*F*(1,141)=0.70, *p*=.405). This suggests that the over exploration in EP was not simply explained by reward learning deficits.

Together, these results suggest that the excessive choice switching in EP, which has been reported in numerous prior studies, was not due to an overall increase in the tendency to switch. Rather, the over-switching was due to a higher probability of transition into fast-switching exploration mode. Participants with EP started exploration too soon when their latest choice was still valuable and exploration provided less new information. What drove participants with EP to transition out of the exploit strategy state and start exploring?

### Model comparison: A Bayesian learner model that adjusts learning rate based on uncertainty estimates explained participant choice behaviors better than RL models with fixed learning rates

Our lack of group differences in probability of switching during exploration and probability of transitioning to exploit strategy suggested that the answer to this question was not simply differences in reward learning. One possibility is that uncertainty associated with unchosen choices could drive a subject to start explorating for information about the environment. We therefore turned to computational models that provide more insight into latent microcognitive processes beyond reward learning that contribute to information processing and decision making.

**Figure 3.**
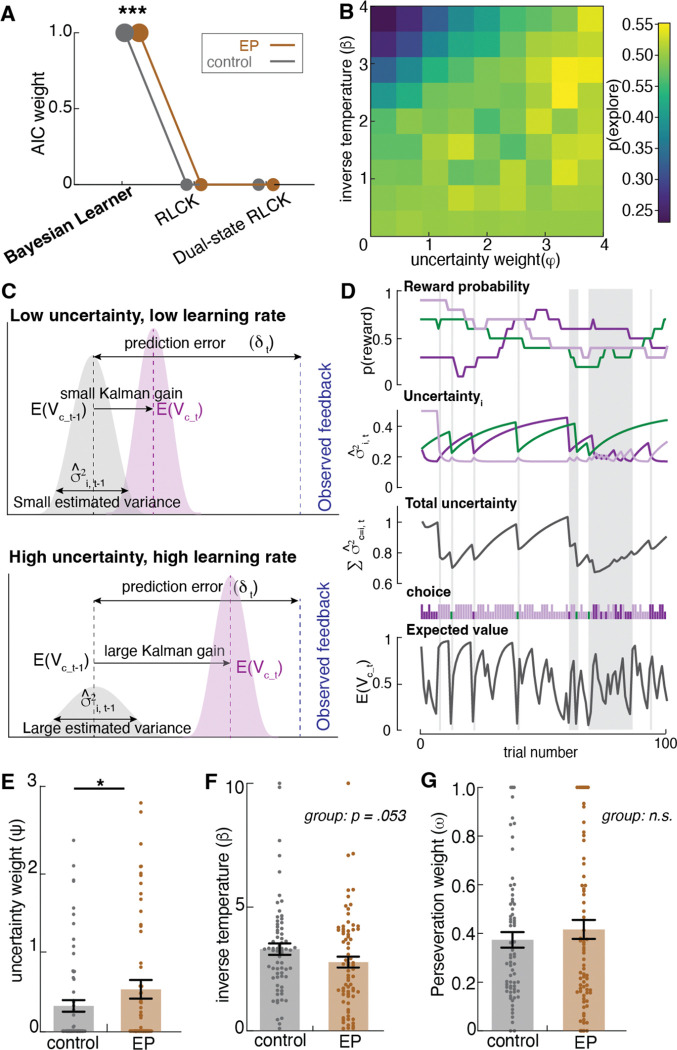
Participants with Early Psychosis had higher decision noise and overweighted uncertainty inferred by a Bayesian learner model, and both processes contributed to over exploration in EP. **A**) Model comparison across three computational models: a Bayesian learner model with Kalman filter, a reinforcement learning-choice kernel (RLCK) model, and a dual-state learning-choice kernel model (dual-state RLCK) model. The Bayesian learner model has the highest relative likelihood (AIC weight) in both controls and EP. **B**) Simulation of 10,000 Bayesian learner agents with different random combinations of inverse temperatures (β) and uncertainty weight (φ), performing the Translation Bandit task and HMMs were fitted to simulated choice sequences to infer the level of exploration. Heatmap of all pairwise combinations of inverse temperature and uncertainty weight. The color of the heatmap represents the level of HMM-inferred exploration. High uncertainty weight or low inverse temperature (high decision noise) leads to a high level of exploration. **C**) An illustration of the adaptive learning rate (Kalman gain) under low and high uncertainty. When uncertainty, captured by the variance of estimated value distribution, is low, the most recent feedback is weighted less and the learned value from reward history is weighted more, less of the prediction error is being updated, the Kalman gain (learning rate) is smaller. When uncertainty is high, the most recent feedback is weighted more, the Kalman gain (learning rate) is larger, which means more of the prediction error is updated. In this model, when uncertainty is high, the model increases learning rate, promoting exploration; when uncertainty is low, the model relies more on learned value, promoting exploitation. **D**) An example of Bayesian Learner model fit to participant data (example of 100 trials) and trial-wise value estimation as well as variance (uncertainty) estimates. The uncertainty level towards an unselected choice gradually increases over time and reduces when being selected. The choice selection is dependent on both expected value and uncertainty of three choices, with some random noise. **E**) Uncertainty weight (φ) fitted to participant choice sequences in Session 1. EPs had higher uncertainty intolerance compared to controls. **F**) Inverse temperature (β) fitted to participants in Session 1. EPs had a trend of lower inverse temperature (higher decision noise) compared to controls. **G**) Perseveration weight (ω) fitted to participants in Session 1. There was no group difference in perseveration weight. Since there were no session effects, only session 1 data is shown here for clarity. Session 2 results can be found in Extended Data Figure 3. * indicates *p* < .05. Graphs depict mean ± SEM across participants.

We first employed a Bayesian learner model that utilized Kalman Filters to fit participants’ choices. Recent work suggests that the Bayesian learning framework is especially well-suited for dynamic tasks like ours, where volatility in reward contingencies challenges individuals to continuously update their beliefs and adapt their strategies^29–32^. This framework explicitly models how individuals adjust their learning rates based on the level of uncertainty in the environment, which has been shown to be relevant in populations with psychosis^6^. Kalman filters adapt the learning rate dynamically based on the uncertainty of the estimates of expected value for each choice in a Bayesian framework (Figure 3C). When uncertainty is high, more weight is placed on new observations (high Kalman gain) and when uncertainty is low, more weight is placed on learned estimates of expected value (low Kalman gain). Due to the dynamic nature of our restless bandit task, a Kalman filter model may allow us to better examine how individuals adaptively updated the estimated reward value as well as the confidence in those estimates. Action selection is dependent on estimated reward value as well as estimated variance (uncertainty) of choices via SoftMax function. Uncertainty weight represents the degree to which uncertainty influences choice selection, and an inverse temperature determines the level of decision noise (See Methods). We also included a choice perseveration term in the Bayesian learner model to account for value-independent choice persistence.

For completeness, we also considered another prominent class of computational models of value-based decision making, that of reinforcement learning (RL). The RL framework describes a process of value updating using feedback^33,34^. RL models are widely used in modeling Bandit task behaviors. However, the learning rates in RL models are often fixed or differ only between specific strategy states. Here, we included RL models as an important comparison because the RL framework provides a robust and well-established baseline to determine whether participants’ decisions can be explained primarily by value-driven learning mechanisms or if there is a unique contribution of uncertainty estimates that the Bayesian approach captures. Therefore, we also fit a standard RL model with a fixed learning rate to participants’ choices, and a dual-state RL model with separate learning rates for the explore and exploit strategy states, as the HMM results showed different sensitivity to feedback during the explore and exploit strategies. Based on our experience and prior literature^26,35–37^, adding a value-independent choice kernel (CK) parameter to capture choice persistence into the RL model significantly improved the modeling of restless bandit choice behaviors, so we incorporated this parameter into the two RL models to capture choice stickiness.

We conducted a model comparison across the three computational models: (1) a three-parameter Bayesian learner model (Kalman filter with uncertainty weight, perseveration weight, and inverse temperature), (2) a four-parameter “RLCK” model (a standard RL model with a single fixed learning rate, a choice kernel, a value-bias scaler, and inverse temperature), (3) a five-parameter “dual-state RLCK” model (a RL model with two different learning rates for explore and exploit state, a choice kernel, as well as inverse temperature parameters for value and for choice). Akaike Information Criterion (AIC) weights of each model for each group were calculated for model comparison to determine the best model with the highest relative likelihood. The three-parameter Bayesian learner model with a Kalman filter was the best-fit model in both the control participant and EP group (Figure 3A). This highlights the importance of an adaptive learning rate that captures dynamic adjustments to changing environmental uncertainty.

### Uncertainty intolerance and decision noise both contribute to changes in exploration-exploitation tradeoff

To determine how changes in the Bayesian learner model parameters influence the level of HMM-inferred exploration, we ran computer simulations of 10,000 Bayesian learner agents defined by different combinations of two main free parameters: uncertainty weight (φ) and inverse temperature (β) doing a three-armed restless bandit task. We fit a Hidden Markov model (HMM) to the simulated choice sequence and inferred each choice as being in an explore or exploit state. Then we plotted the level of exploration produced by agents with varying uncertainty weight and inverse temperature parameters (Figure 3B). As expected, changes in both uncertainty weight and inverse temperature influenced the level of exploration in the restless bandit task. Because higher uncertainty weight reflects greater uncertainty intolerance and lower inverse temperature reflects greater decision noise, we report our results using these descriptive labels throughout (uncertainty intolerance for φ and decision noise for β). Exploration increased with both higher uncertainty intolerance and with higher decision noise (Figure 3B). When uncertainty was weighted more, the drive to seek information to reduce uncertainty about the probabilities outweighed the drive to exploit a known choice for reward, thus leading to exploration. When the decision noise was higher, decision making deviated more away from the learned value of choices, leading to more random exploration across all choices. Figure 3D illustrates an example of a Bayesian Learner model fit to participant data and trial-wise value estimation as well as variance (uncertainty) estimates. Receiving a reward increased the expected value of the selected choice, and the selection of a choice reduced the uncertainty about that choice. But the uncertainty towards the unselected choices gradually increased over time and contributed more weight to choice selection as a result of the increasing information value.

### Participants with early psychosis used the explore strategy more often due to higher intolerance of uncertainty in the environment, along with high decision noise

**Extended Data Figure 3.**
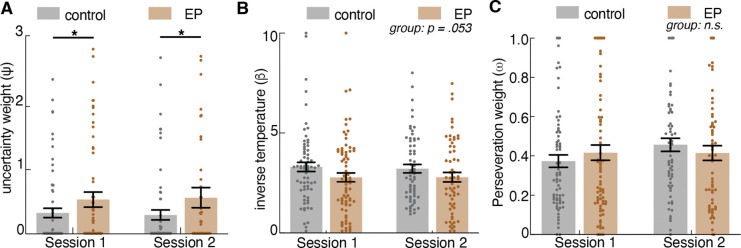
Bayesian learner model parameters across Session 1 and Session 2. Related to Figure 3. **A**) Uncertainty weight (φ) fitted to participant choice sequences in Session 1 and Session 2. **B**) Inverse temperature (β) fitted to participants in Session 1 and Session 2. **C**) Perseveration weight (ω) fitted to participants in Session 1 and Session 2.

To understand what microcognitive processes contribute to the increased exploration in participants with Early Psychosis (EP), we examined the Bayesian model parameters that had been fit to each individual across sessions. Across both sessions, participants with EP exhibited higher uncertainty intolerance during decision making among participants with EP compared to control participants (Figure 3E, Extended Data Figure 3; *F*(141)=7.20, *p*=.008). After Winsorizing two outliers (to the value of the 99^th^ percentile in the distribution; ^38^ ), the effect remained robust (*F*(1,141)=6.20, *p*=.014), indicating that while there were individual differences in model fit, the result was not driven by outliers. Additionally, participants with EP showed a trend toward higher levels of decision noise, suggesting that the decision making of the EP group tended to be less reliant on learned value and that their decision making showed a trend for being more noisy and random, compared to controls (Figure 3F, *F*(141)=3.82, *p*=.053). We found no significant group difference in the perseveration weight parameter across sessions (Figure 3G, *F*(141) = 0.22, *p*=.641), suggesting similar levels of value-independent choice persistence across groups. In summary, the Bayesian learner model revealed that participants with EP explored more due to higher uncertainty intolerance, which drove them to transition to exploration so as to reduce uncertainty by sampling the environment, and high decision noise, which drove more random exploration.

### Uncertainty intolerance and decision noise account for interindividual variance in cognitive processes independent of traditional cognitive measures

**Figure 4.**
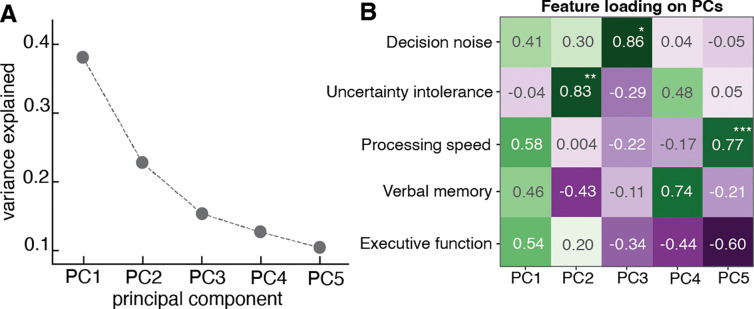
Uncertainty intolerance and decision noise are two independent microcognitive processes that influence explore-exploit tradeoff and are not captured with traditional measures of cognition among participants with early psychosis. **A**) Variance in cognitive processing explained by five orthogonal principal components (PCs). The first three PCs capture the majority of the variance in cognitive processing (~76.5%). **B**) Feature loadings for each principal component. The computational parameters from the restless bandit task (Decision noise, Uncertainty intolerance) captured unique variance from that of traditional cognitive measures (Processing Speed, Verbal Memory, Executive Function from Test My Brain cognitive battery). Significant feature loadings are indicated by * *p*<.05, ** *p*<.01, *** *p*<.001.

In order to determine whether the computational parameters extracted from our task identify relevant cognitive processes beyond traditional measures, we created a vector out of computational parameters from the bandit task (uncertainty intolerance and decision noise) and traditional cognitive scores (processing speed accuracy, verbal memory accuracy, and executive function accuracy) from TestMyBrain measures of processing speed (digit symbol matching test), executive function (matrix reasoning test), and verbal memory (verbal paired associates test) ^39^; we conducted a Principal Component Analysis (PCA) to examine the major axes that capture interindividual variability in cognitive processing. The purpose of the PCA was not to reduce dimensionality but to identify the unique contributions of the various cognitive features to interindividual variability. PCA was applied to these five features (after z-scoring) to identify latent components that capture shared variance. This yielded five interpretable principal components (PCs), with the first three PCs explaining the majority of the variance in cognitive processing (~ 76.5%) (Figure 4A). To examine what each PC represents, we analyzed feature loadings to determine the correlation of each computational and cognitive variable to the principal components (Figure 4B).

The first principal component (PC1) captured ~38% of the variance and loaded moderately on decision noise (loading = 0.41, *p* = .54) and all traditional cognitive measures, including processing speed accuracy (loading = 0.58, *p* = .21), verbal memory accuracy (loading = 0.46, *p* = .47), and executive function accuracy (loading = 0.54, *p* =.29). However, none of these loadings were statistically significant, suggesting that PC1 represents a general cognitive factor encompassing shared variance across multiple domains. The second principal component (PC2), which explained ~23% of the variance, was strongly dominated by uncertainty intolerance (loading = 0.83, *p* = 0.009). Notably, no other feature contributed significantly to PC2. The third principal component (PC3), which explained ~16% of the variance, specifically captured variance related to decision noise (loading = 0.86, *p* = .03) and similarly, traditional cognitive measures contributed minimally to this PC and were not statistically significant. Our findings from PC2 and PC3 indicate that uncertainty intolerance and decision noise each identify a distinct and independent microcognitive process that is orthogonal to traditional cognitive measures. The remaining variance accounted for by PC4 and PC5 was dominated by verbal memory accuracy (loading = 0.74, *p* = 0.051) and processing speed accuracy (loading = 0.77, *p* = .001) respectively, reflecting unique contributions from these cognitive domains. Together, PCA revealed that the computational parameters derived from the task, specifically uncertainty intolerance (PC2) and decision noise (PC3), captured distinct and independent components of interindividual cognitive variance that are not represented by traditional measures of cognitive ability.

### Individual differences in exploratory strategy were linked to non-uniform alterations across microcognitive dimensions

**Extended Data Figure 4.**
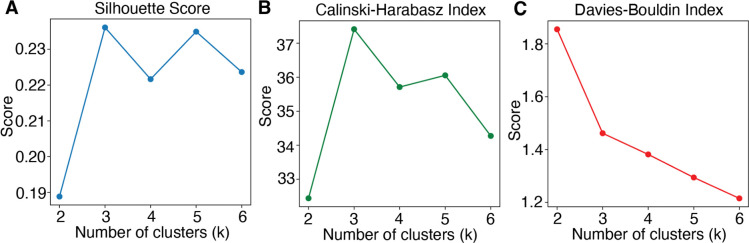
Cluster metrics evaluating cluster separation, cohesion, and overall structure.

**Figure 5.**
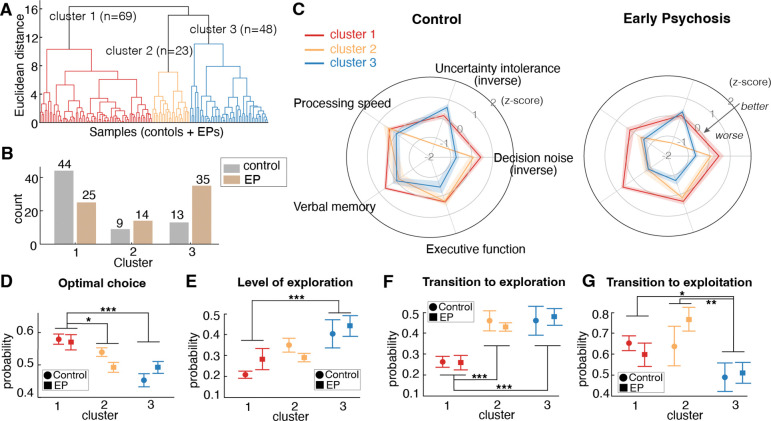
Uncertainty intolerance is a microcognitive process underlying explore-exploit tradeoff that is independent of decision noise and not captured with traditional measures of cognition among participants with early psychosis. **A**) Hierarchical clustering on the computational and cognitive processing measures from session 1 identified three clusters across controls and EPs: 1-red (n=69), 2-orange (n = 23), and 3-blue (n = 48). **B)** Distribution of controls and EPs in each cluster. Controls predominantly occupied cluster 1 while EPs were over-represented in clusters 2 and 3. **C)** Radar plot depicting cluster-specific computational and cognitive profiles in controls and EPs. Each cluster represents a distinct cognitive subtype. Notably, cluster patterns were similar across controls and EPs but more impaired in EPs. Decision noise and uncertainty intolerance were both inverse for the ease of visualization. The more peripheral a cluster, the better cognitive performance and computational stability; the closer to the center, the more impaired the profile. Cluster 1 is characterized by low uncertainty intolerance, low decision noise and strong performance across cognitive measures. Cluster 2 exhibits high uncertainty intolerance but retains relatively intact executive function compared to cluster 3. Cluster 3 shows high decision noise and the most pronounced cognitive impairments across cognitive domains. **D)** Cluster 1 has the highest proportion of optimal choice selections. **E)** Cluster 3 has the highest level of exploration. **F)** Clusters 2 and 3 have the highest probability of transition to exploration. **G)** Cluster 3 has the lowest probability of transitioning to exploitation. Graphs in D-G depict mean ± SEM across subjects, statistics reported in Table 2.

To determine whether variations in these computational and cognitive domains were uniformly altered across all participants or whether they differentiated EPs from healthy controls, we performed hierarchical clustering on the five-dimensional vector of cognitive processing (two computational parameters and three traditional cognitive measures at session 1) using Ward’s linkage method^87^ and examined the profiles and distribution of controls and EPs in each cluster. This approach allowed us to identify three distinct and potentially clinically meaningful computational subtypes among both controls and EPs. Figure 5A shows the dendrogram of the hierarchical clustering structure, and Extended Data Figure 4 shows the cluster metrics.

Although the same three computational subtypes emerged in both groups, their distributions differed: Cluster 1 was predominantly occupied by controls, whereas EPs were over-represented in Cluster 2 and 3 (Figure 5B). We profiled each cluster by calculating the mean values for decision noise, uncertainty intolerance, processing speed, verbal memory, and executive function accuracy (Figure 5C). For ease of visualization in the radar plot, both decision noise and uncertainty intolerance were inverted, so that the more peripheral parameters reflect better cognitive performance and computational stability, whereas closer-to-center parameters indicate more severe impairments. Each cluster characterized a distinct computational subtype. For clarity, we refer to the three clusters as “Subtypes 1–3” throughout. Subtype 1 (predominantly controls) was characterized by low uncertainty intolerance, low decision noise, and the strongest performance across cognitive measures. This indicates a subtype that was normative in its performance across all 5 features. Subtype 2 showed high uncertainty intolerance, low processing speed and low verbal memory yet retained relatively intact executive function. This pattern could suggest a subtype marked by heightened reactivity to uncertainty and slowed information processing. Finally, Subtype 3 was defined by high decision noise (low inverse temperature) and the weakest cognitive performance across all domains. This subtype mirrored prior findings in schizophrenia implicating high decision noise and widespread cognitive impairments ^16^.

Next, we examined how these subtypes differed on task performance and exploration-exploitation dynamics within the entire group of participants. To reduce the risk of inflated Type I errors, all statistical analyses were conducted on the cluster variable rather than on group by cluster, thereby limiting the number of multiple comparisons (Table 2). Subtype 1 (n=69) had the best task performance, indicated by the highest probability of choosing the most optimal choice (Figure 5D). Subtype 3 (n=48) had the highest level of exploration (Figure 5E). Both Subtype 2 (n=23) and Subtype 3 have a higher probability of transitioning to exploration (Figure 5F). Importantly, Subtype 3 had a lower probability of transitioning to exploitation compared to Subtypes 1 and 2 (Figure 5G). This transition dynamic is indicative of reward learning deficits because low probability of transition into exploitation suggests difficulty in learning about the highest valued choice to exploit. This result also highlights that Subtype 2 is distinct from Subtype 3 - though both subtypes over-explored, Subtype 2 did not demonstrate the same reward learning impairments as Subtype 3.

### Exploration and task strategy on the restless Bandit task was associated with clinical features in individuals with Early Psychosis

**Extended Data Figure 5.**
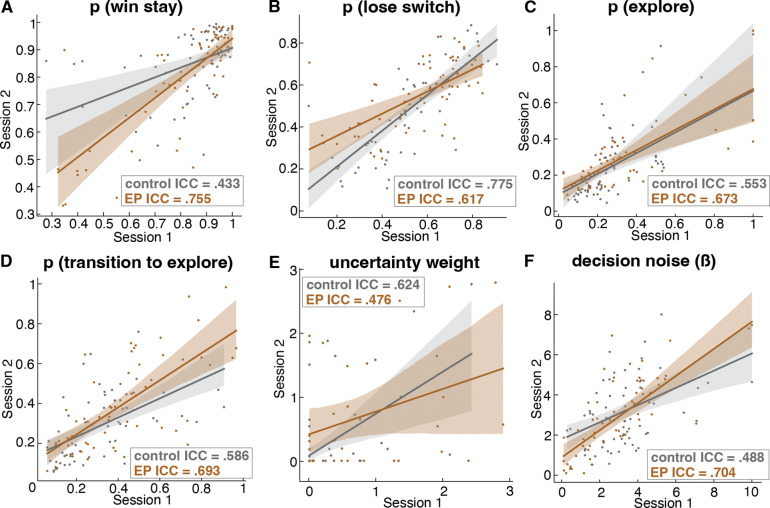
Intraclass Correlation Coefficient (ICC) results revealed moderate test-retest reliability for model-free behavioral parameters and task computational parameters. Task parameters showed moderate reliability using a two-way consistency ICC model for each participant group (control group, n=62, in black, Early Psychosis group, n=60, in blue) across session 1 and session 2, which occurred approximately 3 weeks apart. Each dot represents the correlation, across session 1 and session 2, between a single participant’s probability of engaging in the: **A**) explore strategy state, **B**) maintaining the exploit state strategy, **C**) win-stay strategy, **D**) lose-switch strategy, as well as each participant’s parameter values for: **E**) the Bayesian learner model-derived uncertainty weight parameter, after removal of two outlying data points in the EP group for the purpose of data visualization, and **F**) the Bayesian learner model-derived decision noise parameter. The shaded area represents the 95% confidence interval around the regression line.

The task strategy was consistent across two sessions with little practice effect in both EP and controls, as indicated by moderate test-retest reliability for behavioral parameters reported above. The Intraclass Correlation Coefficient (ICC) results are shown in Extended Data Figure 5.

**Figure 6.**
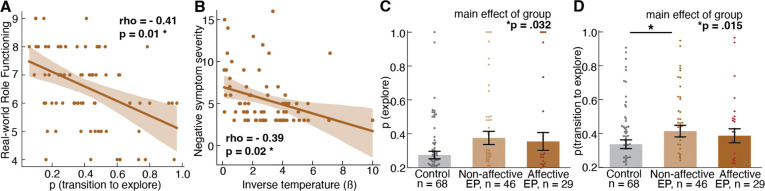
Task strategy was associated with symptom severity and real world functioning among 74 participants with Early Psychosis (EP) at session 1. Symptom severity was measured with the Brief Psychiatric Rating Scale. **A**) Higher probability of transitioning to explore strategy state (fitted HMM transition matrix parameter) correlated with lower real-world functioning. **B**) Lower Bayesian model inverse temperature, indicating higher decision noise, correlated with more severe negative symptom severity. **C**) EP with non-affective psychosis (n=46) and EP with affective psychosis (n=29) had higher probability of exploration compared to controls (n=68). **D)** EP with non-affective psychosis had higher probability of transitioning into exploration strategy state, suggesting altered explore/exploit transition dynamics that is most pronounced in non-affective psychosis. * indicates p < 0.05, ** p<0.01, *** p <0.001.

To test the clinical relevance of the group differences in task strategy within EP individuals, we examined the association between the computational model parameters and standard measures of clinical symptoms and functioning. Specifically, we tested for correlation between 6 clinical variables (negative/ positive/ disorganized/ manic/ depressed symptoms, role functioning) and 3 task variables (transitions to exploration, decision noise, uncertainty intolerance) using Spearman correlations with multiple comparison correction using the False Discovery Rate (FDR) Benjamini-Hochberg procedure (Table 3). Among individuals with Early Psychosis, a higher transition to explore was associated with worse real-world functioning, as measured by the Global Functioning-Role Scale ^40,41^ at session 1 (Figure 6A, *n*=73, *rho* = −0.41, FDR *p* = .014). Additionally, higher decision noise was associated with more severe “negative symptoms” (impaired motivation and lower emotional expressivity), as measured by the Brief Psychiatric Rating Scale-24 Item Version (BPRS; ^42^) (Figure 6B, *n*=74, *rho* = −.39, FDR *p* = .021).

**Extended Data Figure 6.**
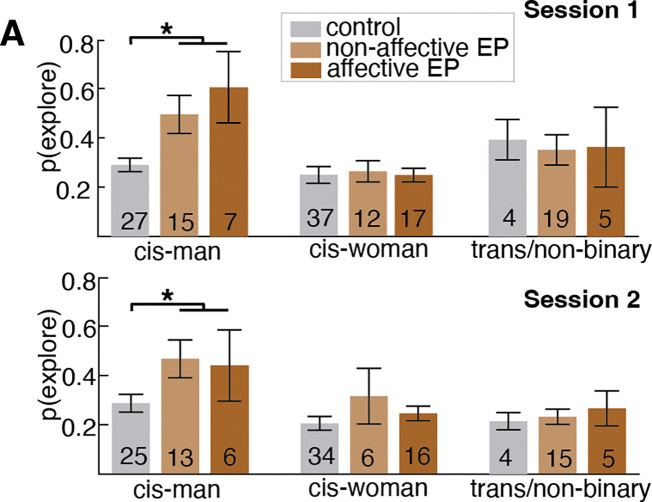
Gender-by-diagnostic group analysis suggested that EP identifying as cis-men exhibited increased exploration. Cis-men with non-affective psychosis and cis-men with affective psychosis had higher probability of exploration compared to controls in Session 1 and Session 2. * indicates p < 0.05, ** p<0.01, *** p <0.001. Graphs depict mean ± SEM across participants.

In an effort to determine whether history of mood dysregulation and resulting diagnostic classification is an important factor in Bandit task strategy, we compared exploration strategy and transition dynamics across controls (*n*=68), non-affective EP (*n*=46), and affective EP (*n*=20; Figure 6C). There was a significant overall effect of group (*F*(2,140)=3.53, *p*=.032), with trend-level posthoc comparisons suggesting that both non-affective (*p*=.062) and affective (*p*=.099) psychosis groups had elevated rates of exploration compared to controls. We also found a significant group difference in transitions to exploration (*F*(2,140)=4.30, *p*=.015) such that non-affective EP had significantly higher transitions to explore than controls (*p*=.019), whereas affective EP were not significantly different from non-affective EP (*p*=.817) or controls (*p*=.136). These results demonstrate that the over-exploration and higher transitions to exploration observed among EP are not driven by EP with a concurrent mood disorder. Additional individual difference analyses are reported in the Supplementary Materials and Extended Data Figure 6.

### Exploratory analysis: Subtypes derived from task parameters were demographically similar but showed unique clinical correlates among individuals with Early Psychosis.

**Figure 7.**
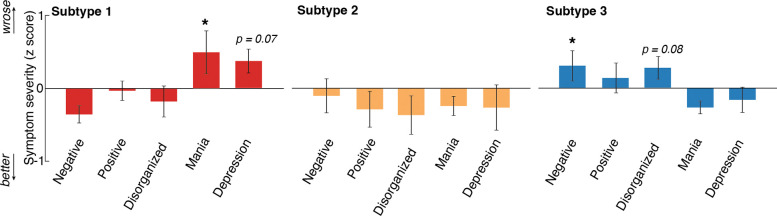
Computational subtypes are associated with different symptom profiles. Symptom severity was measured with the Brief Psychiatric Rating Scale (BPRS), including negative symptom, positive symptom, disorganized symptom, mania and depression symptom subscales. Average symptom severity score was calculated and standardized for each of the three subtypes: subtype 1 - normative, subtype 2 - uncertainty intolerance, subtype 3 - decision noise with cognitive impairments. Graph depicts mean ± SEM across subjects within each subtype. * indicates p<0.05, ** p<0.01, *** p<0.001.

Within the EP sample, computational subtypes differed in terms of negative symptom severity (*F*(2,70)=3.31, *p*=.042) and mania severity (*F*(2,70)=5.06, *p*=.009) over the previous month, as measured by the BPRS ^42^ (Figure 7). Posthoc pairwise comparisons showed that Subtype 3 (decision-noise subtype) had higher negative symptom severity than Subtype 1 (normative subtype) (*p*=.036). Subtype 1 had the highest manic symptom severity compared to Subtype 3 (*p*=.010) and at trend level to Subtype 2 (uncertainty-intolerance subtype) (*p*=.062). There were also trend-level differences in disorganization symptom severity (*F*(2,71)=2.69, *p*=.075) and depression severity (*F*(2,71)=2.72, *p*=.073) across subtypes among EP. To determine whether the differential symptom profiles across computational subtypes linked to other factors, we examined demographic information, diagnostic classification, and psychiatric treatment history across subtypes. There was a significant difference across the computational subtypes in terms of history of multiple psychiatric hospitalizations (*χ*^2^(2)=7.84, *p*=.020) and a trend for diagnostic classification (*χ*^2^(12)=20.63, *p*=.056). Subtype 2 (uncertainty-intolerance subtype) was associated with a higher likelihood of having multiple lifetime psychiatric hospitalizations (adjusted Pearson residual = 2.31). When examining all possible combinations of primary diagnosis and cluster membership, participants with bipolar disorder with psychotic features were over-represented in Subtype 1 (adjusted Pearson residual = 2.90) and under-represented in Subtype 3 (−2.34), whereas participants with schizophrenia were under-represented in Subtype 1 (−2.00). Cluster membership did not differ based on age (*p*=.799), gender (*p*=.118), race (*p*=.16), duration of illness (*p*=.303), or antipsychotic status (*p*=.556).

## Discussion

In this study, we applied computational modeling to a three-armed restless bandit task to characterize how individuals with early psychosis (EP) and controls navigate the tradeoff between exploiting known rewarding choices and exploring alternatives for more information in order to engage in adaptive goal-directed behavior. Despite similar reward acquisition performance, EP were less likely to stick with an optimal choice, which is defined as the highest payoff choice, due to a pronounced tendency to switch choices. This increased choice switching in psychosis is consistent with prior work and has often been explained as due to a reward-learning deficit^6,12–14^. Our results, however, find that the over switching was due to a higher probability of transitioning away from favorable exploitation into exploration among EP, implicating a less stable exploit strategy state. Notably, we found no differences in participants’ propensity to begin exploiting a rewarding option once it was found, suggesting that the over-exploration in EP cannot be explained by the reward learning deficits that prior studies have pointed to. Rather, the excessive choice switching in EP arose from the suboptimal transition into exploration, ending the exploitative phase too soon, even though the previously chosen choice was still favorable, implicating instability, not acquisition, of value-driven exploitative strategy. So why did participants with EP switch to exploring too soon, leaving a favorable option?

### The role of uncertainty intolerance:

Our Bayesian learner modeling suggested that one contributor to this suboptimal shift to exploration was uncertainty intolerance, reflected by increased uncertainty weight during action selection in the computational model. Although decision noise also contributed to the increased exploration in EP (and has been highlighted in prior literature), uncertainty intolerance emerged as a distinct and independent factor that accounted for additional variance in behavior. This is consistent with theories that posit psychosis as a disorder of aberrant inference^6,43–45^. Recent studies have shown that individuals with psychosis inferred the environment as overly volatile, resulting in increased updating to irrelevant information and increased choice switching^6,46–48^. Cole et al. (2020) found that people at clinical high risk for psychosis overestimated environmental volatility and their neural activity showed abnormal increase in prefrontal and insular activation in response to unexpected outcomes, but reduced activation to higher-level changes in belief about volatility. This suggests that uncertainty intolerance may precede illness onset and may have detectable neural correlates that would inform pathophysiological understanding of psychosis.

Previous research with other groups of individuals has also demonstrated variability in behavioral strategies related to uncertainty processing. For example, while some individuals with psychosis respond to uncertainty by excessive exploration, constantly sampling new choices to reduce environmental uncertainty^6,46–48^, others exhibit extreme ambiguity aversion, avoiding uncertain options almost completely^16^. This variability may link to both contextual differences in tasks used to measure explore/exploit decision making^49^ and to individual differences in mood and anxiety conditions, which have been strongly associated with heightened uncertainty intolerance^50–52^ Since psychosis often co-occurs with mood and anxiety symptoms, these factors can collectively shape a person’s cognitive strategy in uncertain environments^53,54^. While affective differences between groups have been clearly documented to correlate with performance in other task contexts, our results suggest that differences between non-affective and affective psychosis groups do not entirely explain decision making instability in early psychosis, suggesting other, perhaps more nuanced, forms of computational failure contribute. Recent reviews have identified neural network correlates, in particular salience network activation during exploration, in psychosis and healthy controls^55,56^. Anxiety-related research has implicated the anterior insula cortex, part of the salience network, during representation of uncertainty^29,57,58^, with several studies reporting hyperactivation of the anterior insula during anticipation of unpredictable or uncertain stimuli in healthy individuals, individuals with high expression of uncertainty intolerance, and individuals with anxiety disorders^59–61^.

### The role of decision noise:

In addition to the role of uncertainty intolerance in over-exploration in EP, our results also implicated elevated decision noise. This is consistent with prior literature indicating increased random exploration/reduced precision in choice (often defined by lower inverse temperature in computational models) leading to more frequent choice switching^62^. A recent meta-analysis demonstrated a pattern of increased switch behaviors in reversal learning and bandit tasks in psychosis across studies, and identified increased decision noise as a key mechanism underlying the increased switching in individuals with schizophrenia^49^. There is some evidence that high decision noise, i.e. random exploration, likely reflects alterations in neuromodulatory systems, in particular dopamine and norepinephrine^20,63^. A recent pharmacological study in mice demonstrated that increasing and decreasing systemic dopamine activity bidirectionally modulated decision noise in a restless bandit task^20^. Noradrenergic tone and related measures like pupil size predict changes in exploration-exploitation tradeoff in healthy humans^64^. Tervo et al. (2014) demonstrated that locus coeruleus input into anterior cingulate cortex controls switching between outcome-based and noise-based behavioral strategies in rats^65^. It is worth noting that the relative influence of “directed” versus “random” exploration can vary across studies, in part stemming from differences in task design and reward structures. Our results suggest that, in EP, both modes of exploration—uncertainty-driven (“directed”) and noise-driven (“random”) exploration contribute to excessive switching and influence the transition dynamics between exploration and exploitation, indicating that random exploration alone does not fully capture the complexity of how individuals with EP deviate from normative explore-exploit dynamics.

### Computational subtyping:

Not all individuals with early psychosis demonstrated the same degree of over-exploration. Hierarchical clustering analysis revealed three distinct computational subtypes within the EP group, each defined by unique explore/exploit transition dynamics and cognition profiles - a normative subtype with low exploration and intact cognition, an uncertainty-intolerance subtype with heightened salience/uncertainty reactivity but no reward learning deficits, and a decision noise subtype with broader cognitive impairments and reward learning deficits. This stratification aligns with the three neurobiologically distinct psychosis biotypes identified by the B-SNIP (Bipolar-Schizophrenia Network on Intermediate Phenotypes) consortium based on biomakers ^66,67^ Biotype 1, characterized by profound dysfunction in cognitive processing and low neural response magnitudes, is consistent with the decision-noise subtype (Subtype 3) in our study. Biotype 2, characterized by overactive neural responses and less severe cognitive control dysfunction, possibly echoes the uncertainty-intolerance subtype (Subtype 2) in our study, which had intermediate cognitive deficits and altered salience processing. Biotype 3, characterized by a near-normal cognitive and sensorimotor profile, is similar to the normative subtype (Subtype 1) in our study. Furthermore, we also replicated the unequal distribution of DSM diagnoses across subtypes. Similar to B-SNIP biotypes, bipolar disorder with psychosis was over-represented in the normative subtype, whereas schizophrenia was more prevalent in Subtypes 2 and 3. These findings highlight heterogeneity in the computational and cognitive functioning of EPs, in line with previously reported neurophysiological heterogeneity, that is not captured by diagnostic classification and thus is missed in clinical settings.

Such heterogeneity underscores the need for precision psychiatry approaches, where different subtypes may benefit from targeted interventions^3^. The alignment with B-SNIP biotypes suggests that future cross study validation may show that a decision making task, paired with fine grained computational modeling, could potentially reproduce the same neurobiological subgroups that required an extensive biomarker panel. More importantly, this alignment may also provide a map to guide mechanism-driven interventions by linking each individual’s microcognitive profile to its putative circuitry. For example, the uncertainty-intolerance subtype may be linked to hyperactivity in the anterior insula-anterior cingulate salience networks and aberrant dopamine signaling, suggesting that interventions like dopamine modulators or targeted cognitive training to improve uncertainty tolerance could be particularly beneficial for this subtype. In contrast, the decision-noise subtype could reflect broad corticostriatal dysfunction and NMDA-receptor hypofunction underlying global cognitive and learning impairments, so NMDAr agonists may show beneficial effects in this subtype. Overall, this computational subtyping approach may offer a more accessible and quantifiable method for precision psychiatry, potentially guiding clinicians for individualized treatment selection.

### Association with clinical features:

The computational task parameters were relatively stable and showed moderate test-retest reliability across two data acquisition sessions (Extended Data Figure 5) despite the dynamic nature of the task. This reliability provides a foundation for examining associations between variations in task parameters and clinical characteristics. Overall, among the full sample of participants with psychosis, we found that symptom severity and real-world functioning were associated with task strategy. Specifically, those with more severe negative symptoms and poorer real-world functioning were less likely to persist in a goal-directed exploit strategy state and more likely to engage in exploratory strategies marked by suboptimal switching and high decision noise. We did not observe significant correlations between psychosis symptoms and uncertainty intolerance on the group level. It is possible that our sample size was not large enough to capture an association, especially given the individual differences within the EP group as seen in the cluster analysis. It is also likely that symptom severity does not map directly to specific microcognitive processes, and that identifying subtypes of patients, transdiagnostically, who engage in different strategies is more important than diagnostic or symptom-based classification for understanding individual differences in decision making and ultimately informing treatment, given the link between cognitive impairment and real-world functioning outcomes such as occupational functioning^68^.

Examining clinical features by computational subtypes revealed more nuanced findings. The clinical correlates of the computational subtypes suggest that a mood disorder prominent subtype (Subtype 1) is characterized by normative learning and explore-exploit dynamics as compared to schizophrenia-spectrum disorder prominent subtypes (Subtype 2 and 3). Further, our results reveal two distinct EP subtypes characterized by over-exploration, but with different underlying computational contributors and clinical correlates - mainly, decision noise and poor reward learning map onto a subtype with elevated negative symptom severity whereas uncertainty intolerance maps onto a subtype with elevated psychiatric hospitalization history, possibly reflecting (perceived) volatility in the person’s experienced environment that may shape how they resolve the explore-exploit dilemma. These findings highlight the nuance of cognitive strategies that computational modeling of task behavior is able to extract, with the potential to improve clinical detection, prediction, and intervention.

### Study limitations:

Several limitations of the study should be noted. First, while we aimed to recruit demographically matched control and EP groups, the gender distribution differed across groups. We therefore included gender as a covariate in our analyses. Second, because reward contingencies in our restless bandit task were randomly generated (within controlled ranges of environmental richness), EP participants experienced slightly lower overall reward availability in the first session by chance. We accounted for this task difference by incorporating reward availability as a covariate in statistical analyses. Extensive behavioral measures across sessions showed no meaningful session differences, suggesting minimal impact on actual decision making behaviors (see Extended Data Figures 1–3). Third, our current cross-sectional design limits evaluation of the long-term stability and predictive validity of computational measures. Lastly, future studies could expand on our findings by assessing a broad range of self-reported traits (e.g., uncertainty tolerance, anxiety) to further enhance understanding of mechanisms underlying heterogeneous decision making behaviors in psychosis.

### Translational Potential:

It is important to highlight the translational and scalable potential of tasks, like the restless bandit, that promote interindividual diversity in decision-making strategies and uncover microcognitive strategies of clinical importance. Explore-exploit decisions are shared cognitive processes across species, making this restless bandit task an ideal tool for translational research across species to link neural mechanisms, pharmacological, genetic, and other potential biological contributors to value-based decision making^26,69,70^. A recent comparative study demonstrated high computational validity of the restless bandit task, showing that humans, non-human primates, and mice all used a combination of fast-switching exploratory and slow-switching exploitive strategies to navigate the task environment^25^. Humans and non-human primates use the same neural circuits, such as the anterior insula and frontoparietal network, to balance value and uncertainty during explore-exploit decisions ^71^. Male and female mice reveal divergent latent explore-exploit strategies even when overall reward acquisition is similar ^26^ and our team recently discovered a novel role of catecholamine neuromodulator activity, in particular dopamine and norepinephrine, in mediating exploration strategy via different underlying cognitive processes^20,26^. The restless bandit in our study carries the potential to be easily ported to human clinical settings to yield insights into aberrant neurocomputational processes warranting intervention as well as animal experiments to yield mechanistic insights. Notably, the computational indices extracted from the task captured processes orthogonal to traditional measures of cognition, including processing speed, verbal memory and executive function. Building on its robust cross-species validity and the fine-grained computational readouts, the restless bandit task can capture each person’s unique transition dynamics between exploration and exploitation in a quantifiable way. By translating mechanistic insights into individualized “decision making fingerprints” with clinical correlates, tasks like this have the potential to guide clinicians towards individually-tailored interventions.

## Methods

### Participants.

Participants were recruited at the University of Minnesota through targeted online advertising (e.g., BuildClinical, community flyers), and, for participants with EP, local psychiatry clinics. A total of 143 participants (75 participants individuals with Early Psychosis (EP) and 68 control participants with no history of psychosis) enrolled in the first session of the study, and 124 participants (n=61 participants with EPs; n=63 control participants) returned for the second session of the study. Participants in the current sample were part of a larger, longitudinal neuroimaging parent study at the University of Minnesota. Inclusion criteria for all participants were: 15–45 years old, English as a primary language, IQ≥70. Exclusion criteria for all participants were: current pregnancy; other MRI contraindications; severe substance use disorder; major neurological disorder; history of clinically significant head injury or significant cognitive training. For control participants, additional exclusion criteria were: current diagnosis or family history of psychotic, bipolar, or autism spectrum disorder. For participants with EP, additional inclusion criteria were: age 15–35 or 35–45 with onset of psychosis spectrum illness in the past 5 years.

Written informed consent, or assent with the consent of a caregiver in the case of minors, was provided by all eligible participants and their capacity to provide informed consent was evaluated prior to study procedures using the University of California Brief Assessment of Capacity to Consent (UBACC) assessment tool ^73^. All procedures were approved by the Institutional Review Board (IRB) at University of Minnesota. Participants completed symptom and functioning assessment (detailed below) as well as the restless Bandit task during fMRI in two separate sessions approximately three weeks apart in order to examine test-retest reliability with a two-way consistency intraclass correlation model. Behavioral data from both sessions were used in analyses; fMRI results will be reported elsewhere. Participants received compensation and bonus payments (up to $15) for task performance each session.

Data from all enrolled participants who completed at least one baseline session were included for data analysis.

### Clinical and Self-report Measures.

After the consent was given, all participants completed a clinical interview and self-report questionnaires that further assessed their symptoms and functioning. Trained research assistants administered the Mini International Neuropsychiatric Interview (MINI), which is a semi-structured diagnostic interview based on DSM-5 criteria, to assess clinical diagnosis in EP and to confirm absence of psychosis diagnosis in controls. In place of the suicidality module of the MINI, the Columbia Suicide Severity Rating Scale screener (C-SSRS) was used because it more succinctly assesses suicidal thoughts and behavior, reducing participant burden. Staff training prior to independent administration of the diagnostic interview involved: reviewing the MINI and C-SSRS user guides and manuals, engaging in a group training and refresher meetings led by a PhD-level member of the team, completing practice ratings of interview videos, completing at least one mock interview with a fellow staff member, observing a trained rater administer at least two diagnostic interviews with study participants, and administering at least two diagnostic interviews with study participants while being observed by a trained rater. Interviewers regularly consulted with a doctorate-level study staff member regarding symptom classification and diagnosis. Age of onset was determined from self-report of symptom and treatment onset as well as review of indicators of functional decline and/or psychiatric hospitalization history. Age of onset was recorded for all participants with EP to calculate duration of illness.

In addition to making diagnostic determinations, we also collected dimensional measures of psychiatric symptom severity. The Brief Psychiatric Rating Scale-24 Item Version (BPRS; ^42^) was collected for both control participants and participants with EP. Within the EP group, psychosis-related symptom severity was assessed using the Scales for the Assessment of Negative/Positive Symptoms (SANS/SAPS; ^74,75^), with sums of global ratings ^76^ reflecting the total negative and positive symptom severity by domain. These measures were used to assess symptoms of psychosis and mood dysregulation regardless of the diagnosis. Ratings on these measures reflect symptom severity over the previous 30 days. Staff training on these measures involved a similar process as the training for diagnostic interview administration (watching introductory videos provided by the scale authors that describe the interview tool, observing and co-rating, being observed by a trained staff member). The most recent inter-rater reliability check yielded an intraclass correlation of 0.92 across ten research staff rating the BPRS measure for a randomly selected sixteen participant interviews. Real-world functioning was measured using clinician ratings of participant engagement and performance in developmentally-appropriate social- and role-related activities at school, work, and home on the Global Functioning Social and Role Scales ^40,41^. Cognitive functioning was assessed using Test My Brain ^39^, which provides measures of processing speed accuracy (digit symbol matching), executive functioning (matrix reasoning), verbal memory accuracy (verbal paired associates), social cognition (multiracial emotion recognition), and an estimate of IQ (derived from performance on verbal paired associates).

### Behavioral Task: three-armed restless Bandit Task.

Participants completed a three-armed restless bandit task ^19^ as part of a larger imaging study. In each trial, participants were presented with three images of Gabor patches (i.e., sinusoidal luminance modulation within a Gaussian envelope, σ = 0.75°) on a mean gray background (Figure 1A). Gabors differed in spatial frequency (0.75, 1.5 or 4 cycles/°) and orientation (45° apart), but were matched for contrast and color. The three Gabor images remained the same throughout the session but the location where each Gabor image appeared was randomized from trial to trial. The three images appeared at 4° eccentricity below-left, above-center, and below-right of the center fixation for 600 ms. Response was indicated by a left, center, or right key press on the task button-box during a 1600 ms response window (starting at the onset of the visual stimuli). The selected image was highlighted by a surrounding box around the selected stimulus for 400–800ms, randomly jittered by 100ms. Then the feedback to the selected choice was provided. The feedback was either +1 point (reward) or +0 points (reward omission) and was shown on the screen for a duration of 300–700ms, randomly jittered by 100ms. We used an empirically validated center fixation mark ^77^, which was also shown during the inter-trial interval with a duration of 500–1500ms, randomly jittered by 200ms.

Each of the Gabor images was associated with some probability of reward, which changed slowly, independently and randomly over time. On each trial, there was a 10% chance that the reward probability would increase or decrease by 0.1 for each arm. The walk (dynamic reward contingency of all three arms) was generated randomly with a few restrictions: (1) to ensure that no arm is constantly high payoff or low payoff, the average payoff probability for each arm across the whole session was required to be 50% ± 2%, (2) to ensure the overall environmental richness was similar across walks, the average payoff probability across all three arms across the whole session was required to be 50%+–2%. (3) the reward probability was bound between 0.1 and 0.9 so the reward contingency was always stochastic and never deterministic (4) the initial reward probability of three arms was fixed at [0.9, 0.7, 0.3] for the first trial to ensure distinctiveness of choices at the beginning. The participants were instructed to collect as many points as possible. The true reward contingency was hidden so the participants could only infer which was the best valued choice through feedback. A 200-trial 3-arm restless Bandit task was administered during functional MRI at 3 Tesla and approximately three weeks later a 300-trial version, each preceded by 25 practice trials, during simultaneous functional MRI and electroencephalogram recording at 3 Tesla. Stimuli were displayed using a BenQ PX9600 projector and viewed on a screen mounted at the back of the scanner through a mirror on the radiofrequency head coil. Projector luminance was linearized using a photometer. A four-button fiber optic response pad (Current Designs Inc. https://www.curdes.com/ Philadelphia, PA, USA) was used to record participant responses. We used a Snellen eye chart ^78^ to measure visual acuity, and provided participants with MRI-safe corrective lenses when needed.

## Data Processing and Analysis

### Statistical techniques.

Data was analyzed using custom PYTHON and R scripts, and Prism 9 and SPSS software. Generalized linear mixed models (GLMMs), ANOVAs, and t-tests were used to determine main effects of clinical groups across both baseline sessions on behavioral measures of task performance. Session and group membership (EP, control) were modeled as fixed effects, participant ID as a random effect, and an interaction term for group × session was added to determine varying slopes representing change over time by group. Covariates included gender and chance level.

Notably, even though the reward contingency (“walk”) was generated and assigned to participants randomly, chance level of reward under each walk differed significantly between groups at session 1 (*F*(1,141)=6.04, *p*=.015) such that random chance of getting a reward was slightly higher for participants with EP (mean = 50.07%, SD=0.75) than control participants (mean = 49.78%, SD=0.65). This would make it slightly more difficult to perform above chance for participants with EP. However, all chance levels of obtaining reward were fixed within a range from 48–52%, inclusive, and chance level of reward was not significantly different between groups at session 2 (*F*(1,122)=0.03, *p*=.860). Thus, this unintentional group difference in walks at session 1, while statistically significant, is not a meaningful difference.

Correlations between task and clinical features were conducted as Spearman’s rho correlations using data from session 1, as clinical data were collected prior to the task session 1 and were not repeated for task session 2. Significance p-values were compared against the standard ɑ = 0.05 threshold, unless otherwise specified. All statistical tests used and statistical details were reported in the results. All figures depict mean ± SEM.

### Reward Sensitivity.

To examine how much of the staying behavior was sensitive to reward outcome vs. reward omission, we calculated the difference between repeating a choice given reward and repeating a choice given no reward, controlled by the total amount of staying. If a participant was not sensitive at all to reward, we would expect to see the reward sensitivity metric close to zero, as the staying behavior was equally likely a result of reward and no reward.


$$\mathrm{reward}\;\mathrm{sensitivity}\;=\frac{p\;\mathrm{(stay|reward)}\;-\;p\;\mathrm{(stay|no}\;\mathrm{reward)}\;}{p\;\mathrm{(stay)}\;}$$

### Mixture of Exponential Distribution.

To examine whether participants’ choice behavior was characterized by a single strategy or multiple strategies with different time constants for switching, we analyzed the temporal structure of the choice sequences using a mixture model of exponential distributions. If choice behavior underlies a single time constant for switching, that is some fixed probability of moving away from the current option at any given trial, then we would expect to see the choice run durations (inter-switch intervals) be exponentially distributed ^24^:

$$f(x)=\beta^{-1}e^{-\frac{x}{\beta }}$$,

where β is the scale parameter, which is related to the average inter-switch interval.

However, if there exists multiple time constants for switching in the choice sequence, the inter-switch intervals would be distributed as a mixture of multiple weighted exponential distributions:

$$f(x)=\sum_{i}^{n}\pi_{i}e^{-\frac{x}{\beta_{i}}}$$,

where $\beta_{i}$ reflects the scale parameter (average inter-switch interval) for each component distribution, and $\pi_{i}$ reflects the relative weight of each component. Therefore, the sum of all weights $\pi_{i}$ is 1. Since inter-switch intervals are discrete, not continuous, we fit mixture models of 1–4 geometric distributions, which are the discrete equivalent of exponential distribution ^79^, via the expectation-maximization algorithm and calculated model log-likelihood for model comparison. Adding a second component distribution significantly improved model fit (Figure 2A, 2B, one component model log likelihood: control: −19233.8, EP: −21039.3, AIC: control: 38618, EP: 42214; mixture model of two components log likelihood: control: −17527.0, EP: −19400.5, AIC: control: 35354, EP: 39073). Although adding a third and fourth component further increased model likelihood (mixture model of three components log likelihood: control:−17440.8, EP: −19281, AIC: control: 35332, EP: 38970; mixture model of four components log likelihood: control: −17432.6, EP: −19274.5, AIC: control: 35465, EP: 39093), the second component granted the highest model performance improvement. Any additional components beyond the second one contributed only marginally to the log likelihood gains ^80^.

In the mixture model fit to controls, the scaling parameter for the fast-switching regime, which is related to the switching time constant, is 1.8 trials, and the relative weight of this component distribution is 0.80; the time constant for the slow switching regime is 8.7 trials, and the relative weight of this component is 0.20. In the mixture model fit to EP, the time constant for the fast-switching regime is 1.7 trials, and the relative weight of this component distribution is 0.85; the time constant of the slow-switching regime is 7.8 trials and the relative weight of this component is 0.15.

### Hidden Markov Model (HMM).

We fit a Hidden Markov Model (HMM) to infer latent states (explore/exploit) from participant choice sequences (Chen et al. 2021; Ebitz et al. 2018). One major assumption of the HMM framework is that choices are generated by some latent unobserved decision processes (“hidden states”) and under each state, there’s some probability of “emitting” a certain choice.

Our model consisted of two types of hidden strategy states, as defined by their unique emission probability of choices as well as transition probability between states. There is one explore state and three exploit states (“exploiting choice 1 state”, “exploiting choice 2 state”, “exploiting choice 3 state”). The exploit states only emit the choice that’s being exploited, e.g.: in exploit choice 1 state, the probability of choosing choice 1 is one, while the probability of choosing choice 2 and 3 is zero. The emissions model for the explore state was uniform across three options because this is the maximum entropy distribution for categorical variables. This does not require, imply, impose, or exclude that choices in explore states are random or structured ^19,26^. The latent states are assumed to be Markovian, meaning that states only depend on the most recent state. The transition matrix describes the one-step transition probabilities between each of the four states. The parameters were tied across the three exploit states, meaning that each exploit state had the same probability of transition into explore state and same probability of staying in exploit state. Transition probabilities from the explore state into one of the three exploit states were also tied. Our model also assumes that to transition from one exploit state to another exploit state, one must pass through the explore state, even just for one trial. Due to the independently changing reward contingency for each choice, when switching from exploiting one option to exploiting another, that very first trial should be exploratory as the reward contingency of the new option was unknown. Similarly, we initialized the first trial of the session in the explore state, as there was no prior knowledge about the reward contingency. The final transition matrix thus has two unique parameters - probability of transitioning from explore state to exploit state, and probability of transitioning from exploit state to explore state.

We fit the HMM model to each participant’s choice sequence using the Baum Welch algorithm ^81^. Fitting the HMM to each participant individually allows us to examine the latent dynamics of states underlying different strategies, and make statistical inferences about the probability that each choice was due to exploration or exploitation.

### Hidden Markov Model State Dynamics.

Further analysis of the transition matrix of the HMM can reveal the dynamics of choice behaviors. Previously, we have utilized analytical tools inspired from approaches used to understand physical mechanics and chemical kinetics to directly characterize the energetic landscape of behavior ^25^. We calculated the stationary distribution of the fitted HMMs, which is the equilibrium probability distribution over explore/exploit states. The stationary distribution is the relative frequency of being in an explore state and an exploit state that we would observe if the model’s dynamics were run for an infinite period of time. Since the Boltzmann distribution gives the probability that a system will be in a certain state as a function of that state’s energy, the stationary distribution of the explore/exploit state allows us to derive the relative energy (depth) associated with each state.

To fully understand the energetic dynamics of the behavior, we also need to understand the activation energy required to transition between states, which is the height of the energetic barrier between two states. The Arrhenius equation relates the rate of transitions away from a state to the activation energy required to escape that state. This equation allows us to calculate the activation energy. Detailed equations can be found in the methods of our previous study ^25,26,28^.

### Bayesian learner model (Kalman filter).

We fit a delta-rule Bayesian learner model that implements the Kalman filter^82–84^ to model adaptive learning and choice selection under uncertainty. The model considered not only estimated value of each choice, but also estimated uncertainty of each choice, along with a perseveration parameter, during the process of choice selection. The Softmax inverse temperature controlled the level of decision noise. The Bayesian learner model assumes that one learns by consistently updating beliefs about the true underlying reward structure of the task - i.e. estimated reward distribution for each choice. On trial t, the model uses the previous feedback $r_{t-1}$ of selected choice $c_{t-1}$ to update the estimated value (EV) as well as the estimated variance $\sigma_{t}$, which reflects the uncertainty/confidence of the value estimates.

Since the restless bandit reward contingency is stochastic (average reward rate across all three arms is 0.5 ± 0.02) and dynamic (change frequency is 0.1, change magnitude is 0.1), we modeled a fixed observation noise parameter ${\overset{ˆ}{\sigma }}_{O}^{2}=0.25$ that represents the stochasticity in observed reward, and a fixed diffusion noise parameter ${\overset{ˆ}{\sigma }}_{D}^{2}=0.1$ that represents the task-specific reward diffusion process to distinguish from random decision noise. Both observation noise and diffusion noise were modeled as a Gaussian distribution with zero mean.

To allow for the learning of a changing reward structure, the model applied a decay parameter λ=0.99 with decay center θ=0.5 to “forget” learned expected value of each choice c on each trial t, according to: $EV_{c,t+1}=\lambda EV_{c,t}+(1-\lambda )\theta$. Similarly, the learned variance estimates also decay according to: ${\overset{ˆ}{\sigma }}_{c,t+1}^{2}=\lambda^{2}{\overset{ˆ}{\sigma }}_{c,t}^{2}+{\overset{ˆ}{\sigma }}_{D}^{2}$. The intuition is that if a certain choice has not been selected over many trials, the learned value estimate of that choice will slowly decay toward random chance level (EV = 0.5), and the confidence in that learned value estimate also decreases, increasing the uncertainty. This is because the structure of the restless bandit task, where the payoff of each choice changes independently and randomly over time.

Participants update their value estimates of the chosen choice according to Bayes’ theorem. On the start of each trial, the prior belief about the reward distribution, which is normally distributed with mean reward rate ${\overset{ˆ}{\mu }}_{c,t}^{\mathrm{pre}}$ and variance ${\overset{ˆ}{\sigma }}_{c,t}^{2\;\mathrm{pre}}$, is updated using prediction error $\delta_{c,t}=r_{t}-{\overset{ˆ}{\mu }}_{c,t}^{\mathrm{pre}}$ by Kalman gain $k_{t}:{\overset{ˆ}{\mu }}_{c,t}^{\mathrm{post}}={\overset{ˆ}{\mu }}_{c,t}^{\mathrm{pre}}+k_{t}\delta_{t}$ and ${\overset{ˆ}{\sigma }}_{c,t}^{2\;\mathrm{post}}=\left(1-k_{t}\right){\overset{ˆ}{\sigma }}_{c,t}^{2\;\mathrm{pre}}$.

The Kalman gain is similar to a learning rate in the reinforcement learning (RL) model in that it determines how much of the prediction error $\delta_{t}$ is being updated for value estimates. However, the Kalman gain depends on the estimated variance of the prior reward distribution and a fixed observation noise: $k_{t}={\overset{ˆ}{\sigma }}_{c,t}^{2\;pre}/({\overset{ˆ}{\sigma }}_{c,t}^{2\;pre}+{\overset{ˆ}{\sigma }}_{O}^{2})$.

The higher the estimated variance of reward distribution for choice c at trial t, the higher the Kalman gain. This means that the learning rate varies from trial to trial and is dependent on the uncertainty/confidence of the value estimate. When uncertainty is high (large estimated variance of reward distribution), the Kalman gain is high so that the immediate past feedback is weighted more and the reward history is weighted less, which promotes more exploration. When uncertainty is low, the Kalman gain is low so that the learned reward value from past trials is weighted more, which promotes more exploitation of a high value choice.

The model also included value-independent perseveration, which is the tendency to repeat the last choice regardless of the previous outcome, weighted by a perseveration weight ω during choice selection. With estimated expected value $\left(EV_{c,t}={\overset{ˆ}{\mu }}_{c,t}^{pre}\right)$, estimated variance $\left({\overset{ˆ}{\sigma }}_{c,t}^{2\;pre}\right)$ and perseveration, the choice selection was performed based on a SoftMax probability distribution:

$$p(c_{t+1}=\frac{e^{\beta (EV_{c,t}+\varphi {\overset{ˆ}{\sigma }}_{c,t}^{2\;pre}+I_{c_{t}=c_{t-1}}\omega )}}{\sum e^{\beta (EV_{c,t}+\varphi {\overset{ˆ}{\sigma }}_{c,t}^{2\;pre}+I_{c_{t}=c_{t-1}}\omega )}}$$

There are thus three free parameters in this model: uncertainty weight φ, perseveration weight ω, and inverse temperature β.φ denotes the uncertainty weight, which represents the degree to which estimated variance (uncertainty) influences choice selection. $I_{c_{t}=c_{t-1}}$ is an indicator that equals 1 when current choice and previous choice are the same and ω is the perseveration weight, which reflects the degree to which the tendency to repeat the previous choice influences choice selection. Finally, inverse temperature β determines the level of random decision noise.

### Reinforcement learning (RL) models.

We fitted two reinforcement learning models that could potentially characterized participants’ choice behaviors. The details of each RL model are described as below:

#### Reinforcement learning + choice kernel (RLCK) model:

this model is a basic delta-rule reinforcement learning model with an additional choice kernel updating rule. The model assumes that one learns by consistently updating an estimated value for each option at each trial $t\;(Q_{t}^{k})$. In each trial, $r_{t}-Q_{t}^{k}$ captures the reward prediction error (RPE), which is the difference between expected value and the actual outcome. The parameter a is the learning rate, which determines the rate of updating RPE.


$$Q_{t+1}^{k}=Q_{t}^{k}+\alpha (r_{t}-Q_{t}^{k})$$

The choice kernel CK captures the value-independent tendency to repeat a biased choice. The choice kernel updating rule is similar to the value-updating rule:

$$CK_{t+1}^{k}=CK_{t}^{k}+\alpha_{c}\left(a_{t}^{k}-CK_{t}^{k}\right)$$

Both value and choice kernel term were combined by a weight τ and used to guide decision making. The action selection was performed based on a Softmax probability distribution,

$$p\left(a_{t+1}=k\right)=\frac{e^{\beta (\tau Q_{t}^{k}+(1-\tau )CK_{t}^{k})}}{\sum_{j}\;(e^{\beta (\tau Q_{t}^{j}+(1-\tau )CK_{t}^{j})})}$$

where the inverse temperature β determines how deterministic or stochastic the choice selection is based on the estimated values and choice kernel. The smaller the inverse temperature beta, the higher the decision noise; the larger the inverse temperature, the more reliance choice selection on estimated value and choice kernel.

#### Dual state reinforcement learning (RL) model.

Since learning rate might be different during explore state and exploit state, we also fitted a dual-state RL model. This model is largely the same as the RLCK model, except this model assumes two learning rates for HMM-inferred explore and exploit state. γ scales learning rate during exploit state.


$$\left\{\begin{array}{c} Q_{t+1}^{k}=Q_{t}^{k}+\alpha (r_{t}-Q_{t}^{k}),\;\mathrm{state}\;=\;\mathrm{explore}\\ Q_{t+1}^{k}=Q_{t}^{k}+\gamma *\alpha (r_{t}-Q_{t}^{k}),\;\mathrm{state}\;=\;\mathrm{exploit} \end{array}\right.$$

AIC values of each model for each group were calculated for model comparison to determine the best model with the highest relative likelihood. Bayesian learner model: AIC = 44472.5, RLCK model: AIC = 45746.4, relative likelihood (AIC weight) of the model <10^−226; dual-state RLCK model: AIC = 45487.1, relative likelihood (AIC weight) of the model <10^−220) and the Early Psychosis group (Bayesian learner model: AIC = 51190.0, RLCK model: AIC = 51684.9, relative likelihood (AIC weight) of the model <10^−178; dual-state RLCK model: AIC = 51430.0, relative likelihood (AIC weight) of the model <10^−52).

### Principal Component Analysis (PCA).

We performed a Principal Component Analysis (PCA) to reduce the dimensionality of all behavioral data and identify key components contributing to variance in the dataset. Five behavioral features were included for analysis: two key computational parameters extracted from the bandit task (uncertainty weight and inverse temperature) and three key cognitive measures from TestMyBrain (processing speed accuracy, verbal memory accuracy, and executive function accuracy). PCA was then applied to the standardized data to ensure all variables contributed equally, irrespective of their original scales. The explained variance ratio for each principal component was calculated to assess the proportion of variance captured. Additionally, the PCA loadings (feature contributions to each principal component) were extracted to interpret the relationship between the original features and the principal components. To assess the significance of feature loadings on each PC, we conducted a permutation test by shuffling the data 1000 times, recalculating the PCA for each permutation, and comparing the observed loadings to null distributions.

### Hierarchical Clustering.

We performed hierarchical clustering based on five cognitive measures (two bandit task-based computational parameters and three traditional cognitive measures from TestMyBrain) using Ward’s linkage method. Only control and EP participants from session 1 were included to avoid repeated measures in the clustering. To determine the optimal number of clusters, we evaluated multiple clustering solutions (number of clusters (k) ranging from 2–6) using three established metrics: Silhouette Score, Calinski-Harabasz Index, and Davies-Bouldin Index. At k = 3, we observed 1) the highest Silhouette Score (0.24), suggesting that best trade-off between separation and compactness, 2) The highest Calinski-Harabasz Index (37.41), indicating the most distinct separation of clusters while maintaining internal cohesion, 3) an elbow in the Davies-Bouldin Index, which measures intra-cluster similarity (Extended Data Figure 4). Based on these convergent results, we selected k=3 because three clusters maximize distinct cluster separation, optimize between-cluster variance, and have high interpretability. The resulting clusters were further examined using original computational and cognitive measures (Radar plot).

## Extended Data

## Supplementary Material

Supplement 1

## Figures and Tables

**Table 1.** Participant Characteristics

Mean (SD) or %	Control n=68	Early Psychosis (EP) n=75	Statistic

**Age**	26.5 (6.8)	25.0 (5.1)	*t*(124.57)=1.47, *p*=.145
**Gender: % cis-woman, cis-man, other**	54.4, 39.7, 5.9	38.7, 29.3, 32.0	*χ*^2^(2,143)=15.46, *p*<.001
**Racial Identity: % White, Black, Indigenous American, Asian, Multiracial**	72.1, 2.9, 0, 16.2, 8.8	70.7, 9.3, 2.7, 6.7, 10.7	*χ*^2^(4,143)=7.15, *p*=.128
**Parent Education, years**	15.5 (3.4)	14.6 (3.0)	*t*(141)= 1.73, *p*=.086
**Estimated IQ**	101.7 (8.3)	99.3 (9.8)	*t*(137)=1.50, *p*=.136
**Symptom Severity, BPRS subscales**			
**Positive symptoms**	5.1 (0.3)	9.9 (5.0)	*t*(74.37)=−8.32, *p*<.0001
**Negative symptoms**	3.6 (1.0)	5.5 (3.2)	*t*(89.19)=−4.96, *p*<.0001
**Disorganized symptoms**	4.9 (1.2)	6.9 (2.0)	*t*(123.9)=−7.34, *p*<.0001
**Depression symptoms**	4.2 (1.5)	8.6 (3.7)	*t*(98.88)=−9.66, *p*<.0001
**Mania symptoms**	3.2 (0.6)	4.0 (1.9)	*t*(90.82)=−3.39, *p*=.001
**Global Functioning, role**	8.5 (0.9)	6.6 (1.4)	*t*(123.18)=10.05, *p*<.001
**Global Functioning, social**	8.5 (1.0)	7.1 (1.6)	*t*(125.85)=6.24, *p*<.001
**Repeat session completed**	92.6%	81.3%	N/A
**Time between sessions (days)**	22.8 (23.6)	23.0 (30.0)	*t*(114.01)=−0.06, *p*=.95
**Diagnoses**	N/A	30.7% schizophrenia, 14.7% schizoaffective, 28.0% bipolar disorder with psychotic features, 10.7% major depression with psychotic features, 16.0% other psychosis	N/A

**Duration of psychotic illness, years**	N/A	5.0 (4.5)	N/A

**Antipsychotic medication load**	N/A	561.4 (1185.6)	N/A

* Note: Gender “other” includes all non-cis gender identities such as trans and non-binary; BPRS = Brief Psychiatric Rating Scale, minimum scores per symptom subscale: positive=5, negative=3, disorganized=4, depression=3, mania=3; Global Functioning ranges from 1(extreme dysfunction) - 10(superior functioning) with 8=good functioning and 7=mild impairment; Diagnoses “other psychosis” category includes other psychosis spectrum disorders such as schizophreniform, psychosis not otherwise specified, and delusional disorder; For analyses, the non-affective psychosis group included diagnoses of schizophrenia, schizoaffective, and other psychosis, whereas the affective psychosis group included bipolar disorder and major depression with psychotic features. Antipsychotic medication load was estimated by calculating chlorpromazine equivalents based on the Defined Daily Dose method ^72^; Missing data due to data loss: 1 control participant and 3 participants with EP on IQ, 1 participant with EP on symptom ratings (BPRS) due to interruption of video function during interview that prevented ratings based on observation, 1 participant with EP on global functioning ratings.

**Table 2.** **ANOVA results and Tukey’s HSD post hoc results for cluster profile** (related to Figure 5D–G)

ANOVA results		

Variable	F-Statistic	p-Value

Optimal choice	13.08	<.001
Level of exploration	12.73	<.001
Transition to explore	19.45	<.001
Transition to exploit	5.98	.003

Tukey’s HSD Results					
Optimal choice (task performance)					

*Group A*	*Group B*	*Mean Diff*	*p-Adj*	*Lower*	*Upper*
1	2	−0.06	**.021**	−0.12	−0.01
1	3	−0.09	**<.001**	−0.14	−0.05
2	3	−0.03	.50	−0.08	0.03
Level of exploration (p(explore))					

*Group A*	*Group B*	*Mean Diff*	*p-Adj*	*Lower*	*Upper*
1	2	0.08	.26	−0.04	0.20
1	3	0.20	**<.001**	0.11	0.29
2	3	0.12	.07	−0.01	0.24
Transition to exploration					

*Group A*	*Group B*	*Mean Diff*	*p-Adj*	*Lower*	*Upper*
1	2	−0.18	**<.001**	−0.29	−0.07
1	3	−0.21	**<.001**	−0.30	−0.13
2	3	−0.03	.79	−0.15	0.08
Transition to exploitation					

*Group A*	*Group B*	*Mean Diff*	*p-Adj*	*Lower*	*Upper*
1	2	−0.08	.38	−0.23	0.07
1	3	0.13	**.028**	0.01	0.24
2	3	0.21	**.005**	0.05	0.36

**Table 3.** **Spearman correlations with FDR-correction for multiple comparisons.** (related to Figure 6)

	1	2	3	4	5	6	7	8	9
*1. BPRS positive*	--								
*2. BPRS negative*	.23	--							
*3. BPRS disorganized*	.56***	.27	--						
*4. BPRS mania*	.30	−.16	.08	--					
*5. BPRS depression*	.42**	.09	.31	.09	--				
*6. Role functioning*	−.21	−.25	−.32	−.09	−.08	--			
*7. Transition to explore*	.01	.34	.03	−.17	−.18	−.41*	--		
*8. Uncertainty intolerance*	−.16	.15	−.18	−.11	−.12	.03	.17	--	
*9. Decision noise*	−.07	−.39*	−.12	.09	.17	.37*	−.73***	.13	--

## Data Availability

The datasets generated and/or analyzed during the current study are available from the corresponding author on request. All data collected as part of this study will be shared with the scientific community. For clinical and behavioral measures, both raw and scaled scores (e.g., estimated IQ; symptom rating measure factors calculated from individual scale items) will be made available. A data dictionary that defines all clinical and behavioral variables will be shared along with the data to aid investigators in selecting their analysis approach. Data will be made publicly available in the NIMH Data Archive (NDA; nda.nih.gov) repository under collection number C3504 upon completion of data collection in 2025.
