# Supplementary material for: Beyond reward learning deficits: Exploration-exploitation instability reveals computational heterogeneity in value-based decision making in early psychosis
Source: medRxiv. 2025 May 1:2025.04.29.25326698. Preprint. [Version 1] doi: 10.1101/2025.04.29.25326698 (PMC12060966; doi:10.1101/2025.04.29.25326698)
Supplement: Supplement 1 [file media-1.pdf]

Supplementary Results.

Results across session 1 and session 2 are qualitatively similar, and are presented for side-by-side comparison in **Extended Data Figures 1-3**. The statistical analysis results presented in the main text account for session time points.

Further analysis of the transition matrix can reveal the dynamics of choice behaviors and provide an intuitive way to visualize the stability of strategy states. We derived the relative energy associated with the explore/exploit state from the stationary distribution of two strategy states and calculated the activation energy required to move from one state to another (See Methods) (**Extended Data Figure 2F**). The stationary distribution of explore/exploit strategy states characterize the stability of each strategy state. The deeper the basin of a state, the more stable the state is. The shallower the state, the less stable it is. The explore and the exploit state each have some inertia, i.e.: some probability of staying in that state. The activation energy of each strategy state characterizes the energy required to transition out of that strategy and into the other strategy. The harder it is to transition out of a strategy state, the higher the height of the energetic barrier. In both control participants and participants with EP, the depth of the exploit state basin was deeper than the explore state, suggesting that exploitation is a more stable strategy state than exploration. Furthermore, in models fit to EP, exploitation was a less stable behavioral state in EP (stationary probability of exploration, EP: 60.0% ± 20.0% STD), compared to models fit to controls (stationary probability of exploration, control: 67.0% ± 16.6%, different from EP: $p=.0024$, 95% CI for the difference = [-11.4%, -2.5%]).

Extended Data Figure 4 shows details of the cluster analysis metrics, suggesting that three separate clusters best represent the data.

Extended Data Figure 5 shows the test-retest reliability of the computational parameters of the task.

We examined whether individual characteristics such as gender identity explained task strategy differences across participant groups. There was a significant group-by-gender interaction effect for probability of exploration (see **Extended Data Figure 6**, $F(2,139)=3.96$, $p=.021$) and uncertainty weighting ($F(2,139)=3.77$, $p=.025$). Pairwise comparisons revealed that cis-men participants with EP had the highest probability of exploration and uncertainty weighting compared to: cis-men control participants ($p<.001$, $p<.001$), cis-women participants with EP ($p<.001$, $p<.001$) and nonbinary participants with EP ($p=.002$; $p<.001$, respectively). We further compared diagnostic classifications and found that cis-men with non-affective psychosis ($p=.007$) and cis-men with affective psychosis ($p=.006$) were more exploratory than cis-men in the control group (**Extended Data Figure 6**). Thus, participants with EP identifying as cis-men had the highest probability of exploration and uncertainty weighting, suggesting that these participants are most likely to engage in the computational failures that are detected by this task.

Given a group by gender interaction effect in predicting probability of exploration, we further investigated individual differences. Interestingly, there was a significant interaction effect between number of lifetime psychiatric hospitalizations and gender for predicting uncertainty weighting ($F(4,135)=2.78$, $p=.030$) such that cis-men participants with EP who had more than one lifetime psychiatric hospitalization had the highest uncertainty weighting compared to cis-men control participants ($p<.001$) and cis-men participants with EP who had one or fewer

hospitalizations (p=.023). This suggests that individual differences in gender identity and lifetime psychiatric hospitalizations, which may represent a proxy for life stressors or volatility in the participant's environment, may contribute to task strategy. The gender effect on exploration was not explained by current symptom severity (BPRS total; $F(2,71)=2.38$, $p=.100$), psychotic illness duration ($F(2,72)=0.33$, $p=.723$), or antipsychotic medication status ($\chi^2(2,75)=5.03$, $p=.081$) when comparing cis-men with EP to other gender identities among participants with EP. Further, there was no evidence of a general pattern of over-exploration among cis-men, based on a non-significant effect of gender on exploration among controls only ($F(2,66)=1.08$, $p=.344$). However, there was a significant gender difference in diagnostic group composition such that cis-women were overrepresented in the affective psychosis group compared to the non-affective psychosis group among EP ($\chi^2(2,75)=8.52$, $p=.014$). We therefore compared affective vs. non-affective psychosis vs. control groups and found a gender-by-diagnostic group interaction effect trending towards significance for probability of exploration (see **Extended Data Figure 6**, $F(4,136)=2.09$, $p=.085$) such that cis-men with non-affective psychosis (p=.007) and cis-men with affective psychosis (p=.006) were more exploratory than cis-men in the control group. Within the non-affective psychosis group, cis-men were more exploratory than cis-women (p=.013) and nonbinary participants (p=.023). Within the affective psychosis group, cis-men were more exploratory than cis-women (p=.002) and nonbinary participants (p=.092). We also found a significant gender-by-diagnostic group interaction for uncertainty weighting ($F(4,136)=2.73$, $p=.032$) such that cis-men with non-affective psychosis (p=.032) and cis-men with affective psychosis ($p<.001$) had a higher uncertainty weighting than cis-men in the control group. Within the non-affective psychosis group, cis-men had a higher uncertainty weighting than nonbinary participants (p=.007). Within the affective psychosis group, cis-men had a higher uncertainty weighting than cis-women ($p<.001$) and nonbinary participants (p=.003). Notably, antipsychotic medication status was equally distributed across affective and non-affective groups ($\chi^2(1,74)=0.13$, $p=.719$). Finally, there was no significant effect of age, or age-by-group interaction, on computational task parameters ($p>.409$). Together, these results suggest a link between individual differences in task strategy, gender identity, and lifetime psychiatric hospitalizations that was not driven by clinical characteristics (symptom severity, duration of psychotic illness, diagnostic subgroups, antipsychotic medication) among participants with EP.

Supplementary Discussion. Examining the influence of gender groups and clinical characteristics adds another layer of complexity. We found that over-exploration was driven by participants with EP who identified as cis-men, regardless of whether in the affective or non-affective psychosis group. We also found that overweighting of uncertainty, which partially explained group differences in exploration, was driven by participants with EP with multiple hospitalizations as compared to those with one or none. Thus, individual differences in gender identity and lifetime psychiatric hospitalizations, which may represent a proxy for life stressors or volatility in the participant's environment, may contribute to task strategy. This is consistent with previous work suggesting that there are subtypes of people with psychosis who have different patterns of exploration[16]. Our participants were relatively young, with relatively recent psychosis onset (median duration of psychosis was three and a half years). Previous research suggests that adolescence is characterized by enhanced novelty seeking and response shifting especially after negative feedback[85]. Further, directed, information-seeking, exploration increases during typical development in adolescence[86]. It is possible that participants with EP in our

sample have not yet matured past this stage of typical development. Alternatively, it is possible that their development of more adaptive strategies was interrupted by the illness onset during adolescence/early adulthood. We did not observe an effect of age, or an age-by-group interaction, on computational task parameters, suggesting the latter interpretation. This implies that participants with EP have an altered developmental trajectory of value-based decision making strategy that warrants clinical attention. Future longitudinal work is needed to determine how task strategy changes over time and with varying clinical trajectories and interventions during this early, neuroplastic phase of illness.
